# Delayed transplantation of precursor cell-derived astrocytes provides multiple benefits in a rat model of Parkinsons

**DOI:** 10.1002/emmm.201302878

**Published:** 2014-01-29

**Authors:** Christoph Proschel, Jennifer L Stripay, Chung-Hsuan Shih, Joshua C Munger, Mark D Noble

**Affiliations:** 1 Department for Biomedical Genetics, University of RochesterRochester, NY, USA; 2 Stem Cell and Regenerative Medicine Institute, University of RochesterRochester, NY, USA; 3 Neuroscience Graduate Program, University of RochesterRochester, NY, USA; 4 Department of Pathology, University of RochesterRochester, NY, USA; 5 Department of Biochemistry and Biophysics, University of RochesterRochester, NY, USA

**Keywords:** astrocytes, cell therapy, neurodegeneration

## Abstract

In addition to dopaminergic neuron loss, it is clear that Parkinson disease includes other pathological changes, including loss of additional neuronal populations. As a means of addressing multiple pathological changes with a single therapeutically-relevant approach, we employed delayed transplantation of a unique class of astrocytes, GDAs^BMP^, that are generated *in vitro* by directed differentiation of glial precursors. GDAs^BMP^ produce multiple agents of interest as treatments for PD and other neurodegenerative disorders, including BDNF, GDNF, neurturin and IGF1. GDAs^BMP^ also exhibit increased levels of antioxidant pathway components, including levels of NADPH and glutathione. Delayed GDA^BMP^ transplantation into the 6-hydroxydopamine lesioned rat striatum restored tyrosine hydroxylase expression and promoted behavioral recovery. GDA^BMP^ transplantation also rescued pathological changes not prevented in other studies, such as the rescue of parvalbumin^+^ GABAergic interneurons. Consistent with expression of the synaptic modulatory proteins thrombospondin-1 and 2 by GDAs^BMP^, increased expression of the synaptic protein synaptophysin was also observed. Thus, GDAs^BMP^ offer a multimodal support cell therapy that provides multiple benefits without requiring prior genetic manipulation.

## Introduction

Developing treatments for neurodegenerative diseases poses a complex therapeutic challenge, with Parkinson's disease (PD) offering an excellent example of the complexity of pathological changes that need to be addressed. Although PD is best known for the degeneration of dopaminergic neurons that originate in the substantia nigra and innervate target neurons in the striatum, individuals with PD show a much broader range of changes which are believed to play important roles in PD disease pathology (Lang & Obeso, 2004). For example, progressive loss of additional neuronal populations such as GABAergic interneuron populations also are thought to contribute to neurological deficits in PD (Kleppner & Tobin, 2001). The absence of appropriate dopaminergic innervation, coupled with damage to other neuronal circuitry and changes in synaptic proteins (Zhan *et al*, 1993; Nishimura *et al*, 1994; Wakabayashi *et al*, 1994; Huynh *et al*, 2003), results in an imbalance between excitatory and inhibitory projections to the cortex and impaired motor control.

While an increasing repertoire of experimental therapies have become available for potential treatment of PD (Olanow *et al*, 2009), the vast majority are focused on either preventing further loss of dopaminergic nigrostriatal neurons by pharmacological intervention or else transplanting new dopaminergic neurons into the striatum itself (Hauser, 2011; Jankovic & Poewe, 2012). A variety of substances that promote dopaminergic neuron survival have been identified, with glial-derived neurotrophic factor (GDNF), brain-derived neurotrophic factor (BDNF) and neurturin being the most extensively studied in these regards (Kirschner *et al*, 1996; Rosenblad *et al*, 1998; Andereggen *et al*, 2009; Herzog *et al*, 2009; Yang *et al*, 2009; Allen *et al*, 2013). These substances have been delivered with implantable infusion devices, viral vectors and transplantation of genetically modified cells in experimental models of PD (Cunningham & Su, 2002; Mohapel *et al*, 2005; Lang *et al*, 2006; Sadan *et al*, 2009; Biju *et al*, 2010; Marks *et al*, 2010). In addition, attempts to reduce oxidative stress, a key mediator of neuronal loss in both familial and idiopathic PD, also results in improved outcomes (Jakel *et al*, 2007; Zhou *et al*, 2008). Such strategies represent an alternative to the transplantation of dopaminergic neurons, which although providing benefit to some individuals also has been associated with the generation of tardive dyskinesias (Lane & Winkler, 2012). Strategies targeted at neuronal protection also provide an important alternative to both dopamine replacement therapy and deep brain stimulation (Bronstein *et al*, 2011), which may provide only a transient benefit targeted primarily at symptom management.

Although rescue of dopaminergic neurons is a critical goal, the additional damage that occurs in PD remains unaddressed and it remains necessary to discover therapeutic approaches that simultaneously provide multiple therapeutic benefits. We now provide such a therapeutic approach based on transplantation of a novel population of astrocytes derived from a well-defined population of embryonic glial-restricted precursor (GRP) cells by exposure to bone morphogenetic protein (designated as GDAs^BMP^). We previously found that acute transplantation of GDAs^BMP^ into the injured spinal cord promoted survival of multiple neuronal populations, axonal regeneration and behavioral recovery (Davies *et al*, 2006, 2011). In contrast, transplantation of astrocytes derived from GRP cells by exposure to ciliary neurotrophic factor (designated as GDAs^CNTF^), or of GRP cells themselves, provided no benefit (Davies *et al*, 2008). Among the multiple ways that GDAs^BMP^ and GDAs^CNTF^ differed from each other, GDAs^CNTF^ resembled reactive astrocytes in several ways, including high expression of glial fibrillary protein (GFAP) and increased expression of chondroitin sulfate proteoglycans (CSPGs)(Davies *et al*, 2011). We further found that GDAs^BMP^ produced elevated levels of brain-derived neurotrophic factor (BDNF) and glial-derived neurotrophic factor (GDNF), two neurotrophic factors of particular interest as potential interventions for therapy of PD (Altar *et al*, 1992; Sauer *et al*, 1994; Yurek *et al*, 1996; Rosenblad *et al*, 1999; Kirik *et al*, 2001; Wang *et al*, 2002; Ben-Hur *et al*, 2004; Sun *et al*, 2005; Saylor *et al*, 2006).

We now report that delayed transplantation of either rat or human (h)GDAs^BMP^ cells into 6-OHDA hemi-lesioned rats after the emergence of motor symptoms resulted in normalized behavior and restored levels of tyrosine hydroxylase (TH) expression. More detailed analysis revealed that hGDA^BMP^ transplantation also rescued parvalbumin-positive GABAergic interneurons, a population of cells that is lost in PD but has not been rescued by any prior experimental interventions. In addition, hGDA^BMP^ transplants also restored expression of synaptophysin, a protein critical in normal synaptic function. In contrast with previous attempts to develop astrocyte-based therapies using cells genetically engineered *in vitro* (Lundberg *et al*, 1996; Cunningham & Su, 2002; Ericson *et al*, 2005; Jakel *et al*, 2007; Sandhu *et al*, 2009; Drinkut *et al*, 2012), GDAs^BMP^ do not require genetic modification to provide benefit as they intrinsically produce multiple molecules of relevance to PD treatment. Thus, we now provide a single therapeutic approach using human cells that promotes behavioral recovery, rescues both dopaminergic and parvalbumin-expressing neurons and increases expression of synaptophysin in an experimental model of PD.

## Results

### GDAs^BMP^ produce multiple agents relevant to PD therapy and rescue dopaminergic neurons from oxidative stress *in vitro*

**Figure 1:**
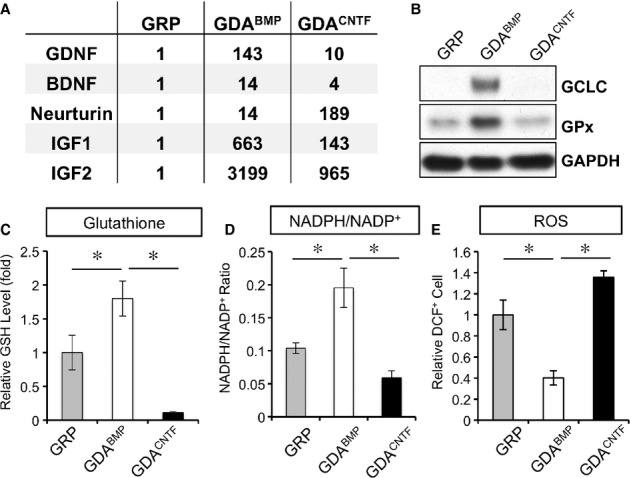
GDAs^BMP^ produce multiple neurotrophic factors and antioxidants. A Gene expression analysis of neurotrophic factors by semi-quantitative RT-PCR shows marked elevation in expression of multiple trophic agents. Expression normalized to expression in GRP cells. Mean of *n* = 4, *P* < 0.05 for all pairwise comparisons by One-Way ANOVA and Bonferroni Multiple Comparison Test except for: GRP versus GDA^CNTF^ for GDNF, BDNF, IGF1 and GRP versus GDA^BMP^ for Neurturin. B Western blot analysis of antioxidant response pathway enzymes: GDAs^BMP^ express increased levels of γ-glutamylcysteine synthetase (γGCS) and glutathione peroxidase (GPx). C–E GDAs^BMP^ show increased levels of glutathione and NADPH/NADP^+^ and lower levels of reactive oxygen species.. Mean ± s.e.m., *n* = 3, *P < 0.05 by ANOVA/ Bonferroni Multiple Comparison post-test.

As one of the major avenues of therapeutic investigation in PD has been the delivery of candidate therapeutic agents, and we previously observed increased production of two such candidates (GDNF and BDNF) by GDAs^BMP^ (Davies *et al*, 2008, 2011), we first examined whether generation of GDAs^BMP^ from rat GRP cells is associated with the expression of other agents of potential interest in PD treatment (Fig 1A). We found that the generation of GDAs^BMP^ from GRP cells was associated with a dramatic increase in mRNA levels for GDNF, BDNF, IGF1 and neurturin, all of which are of interest as potential therapeutic agents in PD. In addition, GDAs^BMP^ also expressed high levels of IGF2. With the exception of neurturin, differentiation of GRP cells into astrocytes was in and of itself not sufficient to induce these dramatic increases in neurotrophic factor expression, as astrocytic differentiation of GRP cells by exposure to ciliary neurotrophic factor (GDAs^CNTF^), was associated with a lesser extent of expression of mRNA for these factors. This is consistent with our previous observations that demonstrate marked phenotypical and functional differences between these two distinct astrocyte populations (Davies *et al*, 2006, 2008, 2011).

Generation of GDAs^BMP^ also was associated with changes predictive of an improved ability to protect against oxidative damage, which is thought to play an important role in the pathogenesis of PD. These cells showed marked increases in levels of GCLC protein, the catalytic domain of γ-glutamylcysteine synthetase (γGCS), and of glutathione peroxidase (GPx), which plays a key role in reducing lipid peroxides (Spina & Cohen, 1989) (Fig 1B). Consistent with the increased expression of GCLC, GDAs^BMP^ showed a two-fold increase in levels of glutathione (Fig 1C) and in the ratio of reduced to oxidized pyridine nucleotides (i.e. NADPH:NADP^+^) as compared with GRP cells (Fig 1D). GDAs^BMP^ also showed a 60% reduction in levels of reactive oxidative species (ROS) as compared with GRP cells (Fig 1E). In contrast, GDAs^CNTF^, did not show equivalent changes in expression of GCLC, GPx, GSH, ROS or in the ratio of reduced to oxidized pyridine nucleotides. Levels of GSH in GDAs^CNTF^ were only 6% those found in GDAs^BMP^, while the levels of ROS were twice as high in GDAs^CNTF^.

**Figure 2:**
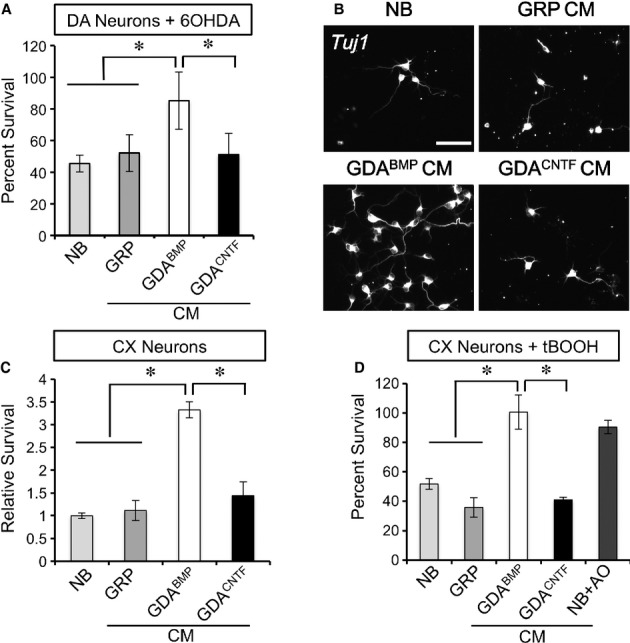
GDAs^BMP^ promote neuronal survival in *vitro*. A GDA^BMP^-conditioned medium (CM) rescued rat midbrain dopaminergic neurons from 10 μM 6-OHDA toxicity,, while GRP-or GDA^CNTF^-CM was no more effective than neurobasal (NB) medium. Mean ± s.e.m., *n* = 3. *P < 0.05 by ANOVA/ Bonferroni Multiple Comparison post-test. B, C Quantification of relative survival after 48 h shows support of cortical (CX) neurons by GDA^BMP^-CM, while GRP-or GDA^CNTF^-CM was no more effective than neurobasal (NB) medium. Mean ± s.e.m., *n* = 3. *P < 0.05 by ANOVA/ Bonferroni Multiple Comparison post-test. D Survival of cortical neurons in neurobasal medium (NB), GRP-, GDA^BMP^-or GDA^CNTF^-conditioned medium (CM) after addition of 1 μM tBOOH. Both GDA^BMP^-CM and addition of antioxidants (AO) protect against tBOOH. Mean ± s.e.m., *n* = 3. *P < 0.05 by ANOVA/ Bonferroni Multiple Comparison post-test.

As predicted from the multiple beneficial factors produced by GDAs^BMP^, these cells were effective at promoting survival of TH^+^ neurons and at protecting these cells from oxidative stress induced by exposure to 6-OHDA *in vitro*. Primary rat midbrain dopaminergic neurons were placed in minimal growth conditions supplemented with conditioned media (CM) from GRP cells, GDAs^BMP^ or GDAs^CNTF^ and challenged with 10 μM 6-OHDA (Fig 2A). While CM from GRP cells or GDAs^CNTF^ showed no benefit, GDA^BMP^-derived CM was able to promote survival of 80% of TH^+^ neurons.

GDAs^BMP^ were also effective at promoting survival of other neuronal populations and protecting them from oxidative stress. In these experiments, rat cortical neurons were placed in minimal medium supplemented with CM from GRP cells, GDAs^BMP^ and GDAs^CNTF^. After 24 h, cortical neuron survival in GDA^BMP^-CM was over three times greater than in control medium, GRP-or GDA^CNTF^-CM (Fig 2B and C). GDA^BMP^-CM, but not CM from GRP cells or GDAs^CNTF^, also rescued cortical neurons from tert-butyl hydroperoxide (tBOOH), a potent pro-oxidant, and was equally as effective as addition of antioxidant supplements (Fig 2D). GDA^BMP^-CM also protected cortical neurons from the toxicity of 6-OHDA (Supplementary Fig 1).

### Delayed transplantation of GDAs^BMP^ into the 6-OHDA hemiparkinsonian rat model restores levels of tyrosine hydroxylase expression

Previous studies on means of rescuing experimental animals from the effects of 6-OHDA injection demonstrated that BDNF or GDNF, delivered from genetically modified cells, by AAV injection or by infusion, can rescue levels of TH in the striatum when administered post-injury (Levivier *et al*, 1995; Klein *et al*, 1999; Rosenblad *et al*, 1999; Kirik *et al*, 2001; Cunningham & Su, 2002; Wang *et al*, 2002; Cohen *et al*, 2003; Sun *et al*, 2005; Yasuhara *et al*, 2005; Ebert *et al*, 2008; Madhavan *et al*, 2009; Sadan *et al*, 2009; Glavaski-Joksimovic *et al*, 2010; Somoza *et al*, 2010; Khoo *et al*, 2011). In addition, astrocytes genetically modified to increase their levels of Nrf2 expression (thus increasing anti-oxidant function) also reduced lesion volume, based on TH immunocytochemistry, when transplanted 5 weeks prior to injection of 6-OHDA. This is in contrast with a lack of effect after transplantation of unmodified astrocytes (Lundberg *et al*, 1996; Jakel *et al*, 2007). The clinical need, however, is to develop therapies effective after symptoms already exist, a challenge for which GDAs^BMP^ were found to be well suited.

We found that delayed transplantation of GDAs^BMP^ provides the reported benefits obtained with delivery of GDNF or BDNF or by transplantation of genetically modified support cells. These experiments employed the unilateral 6-OHDA-injection model, the most extensively characterized model of progressive PD. Adult Fisher 344 rats received three injections of 6-OHDA into the right-side pars compacta of the striatum, a paradigm in which neuronal loss occurs over several weeks (Perese *et al*, 1989; Berger *et al*, 1991; Sauer & Oertel, 1994; Deumens *et al*, 2002).

**Figure 3:**
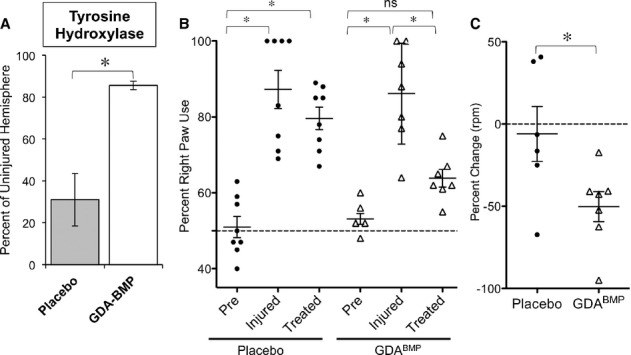
Delayed transplantation of GDAs^BMP^ into the hemiparkinsonian rat model restores levels of tyrosine hydroxylase expression and promotes functional recovery.
Quantification of TH labeling in striatum of placebo and GDA^BMP^ treated animals shows restoration of labeling to levels seen in the uninjured hemisphere. Mean ± s.e.m., *n* = 3. *P < 0.05, Unpaired *t*-test.Preferred right paw use after unilateral 6-OHDA lesion as also restored to near-baseline levels by GDA^BMP^ transplantation. Paw usage was determined for each treatment group prior to injury (Pre), 2 weeks after injury (Injured) and 2 weeks after treatment (Treated). Mean ± s.e.m., *n* = 8 for placebo, *n* = 7 for GDA^BMP^ treated group. *P < 0.05 by ANOVA/ Bonferroni Multiple Comparison post-test.GDA^BMP^ treatment improves rotational response to amphetamine injection. Percent change of rotations per minute before and after treatment for placebo and GDA^BMP^-treatment group. Mean ± s.e.m., *n* = 6. *P < 0.05, Unpaired *t*-test.

Despite the fact that we transplanted astrocytes and not neurons, we found that delayed transplantation of rat GDAs^BMP^ into the injured striatum rescued TH expression to such an extent that levels of TH expression in transplanted striatum were not significantly different from unlesioned striatum (Fig 3A). Twenty-four days after GDA^BMP^ transplantation, animals were sacrificed and coronal sections of the striatum were labeled with antibodies against TH. Co-labeling with ED1 (anti-CD68), a marker of infiltrating mononuclear cells that populate the lesion site, was used to identify the injured side in this unilateral 6-OHDA model. For each section, TH expression was normalized to the contralateral, uninjured side. Seven weeks after injection of 6-OHDA, we found a 70% reduction in TH levels on the injured side in placebo treated animals, while animals receiving a GDA^BMP^ transplant 3 weeks earlier exhibited levels of TH statistically indistinguishable from the uninjured striatum. (For an overview of experimental timeline, see Supplementary Fig 3).

### Delayed transplantation of GDAsBMP into the 6-OHDA model of PD promotes functional recovery

Based on the ability of delayed GDA^BMP^ transplantation to rescue TH expression in the striatum, we asked whether these cells also promoted behavioral recovery. Two weeks after 6-OHDA-injection, motor impairment was determined by Cylinder test, to measure preference of forepaw usage, and D-metamphetamine-induced rotational behavior. Animals with motor defects were randomly assigned to two cohorts. Using the same coordinates as for the 6-OHDA injections, one cohort (“placebo”) received three injections of saline, while the second cohort (“GDA^BMP^ Treated”) received three injections of 1 × 10^5^ syngeneic GDAs^BMP^ each. These cells also expressed cytoplasmic human placental alkaline phosphatase (Kisseberth *et al*, 1999), and were readily detected by immunostaining more than 3 weeks after transplantation.

Rats examined at 14 days post 6-OHDA-injection showed significant changes in paw usage. Lesioned animals exhibited on average a > 85% preference for the right paw, as contrasted with an unbiased 50% right paw use pre-injury (Fig 3B). While this motor deficit persisted in saline treated animals, animals that received GDA^BMP^ transplants 4 weeks after 6-OHDA injection demonstrated a significant improvement in forepaw usage (*P* < 0.05, Fig 3B).

Susceptibility to amphetamine-induced rotational behavior was also significantly reduced in rats receiving delayed GDA^BMP^ transplants (Fig 3C, *P* < 0.05). After 6-OHDA lesioning, all animals showed evidence of amphetamine-induced rotational behavior, exhibiting ipsilateral rotation rates of approximately 10 turns per min. In animals that received intrathecal saline injections (placebo group) no improvement was seen between 2 weeks post-lesion (i.e. 2 weeks before intervention) and 6 weeks post-lesion (i.e. 3 weeks after intervention). In contrast, rats transplanted with GDAs^BMP^ showed a significant (50%, *P* < 0.034) decrease in rotational behavior as compared with the behavior pre-transplantation.

### Delayed transplantation of human fetal cell-derived GDAs^BMP^ into the 6-OHDA model of PD restores levels of TH and promotes functional recovery

As any clinical application of this strategy will require transplantation of human cells, we next examined whether delayed transplantation of human-derived GDAs^BMP^ (hGDAs^BMP^) (Davies *et al*, 2011) was as effective as transplantation of rat cells in rescuing the neurological deficits in 6-OHDA-lesioned rats. Experimental interventions and treatments were performed at time points and as described for transplantation of rat-derived GDAs^BMP^ (Supplementary Fig 2). Transplanted hGDAs could readily be detected by labeling with human specific mitochondrial antigen (Supplementary Fig 4).

**Figure 4:**
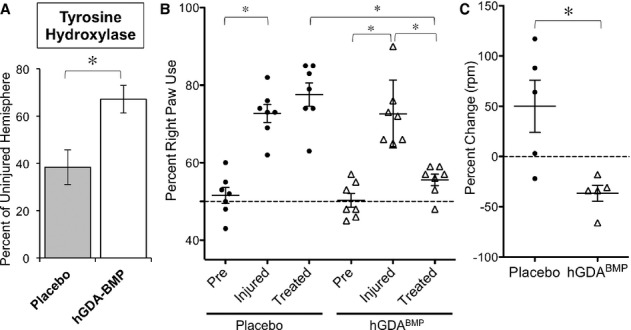
Delayed transplantation of human GDAs^BMP^ into the hemiparkinsonian rat model restores tyrosine hydroxylase expression and promotes functional recovery.
Quantification of TH labeling in striatum of placebo and hGDA^BMP^ treated animals shows significant rescue of TH expression in transplanted tissue. Relative TH expression compared between injured and uninjured hemispheres. Mean ± s.e.m., *n* = 3. *P < 0.05, Unpaired *t*-test.Preferred right paw use after unilateral 6-OHDA lesion recovers to near-baseline levels following GDA^BMP^ transplantation. Paw usage was determined for each treatment group prior to injury (Pre), 2 weeks after injury (Injured) and 2 weeks after treatment (Treated). Mean ± s.e.m., *n* = 7 for both groups. *P < 0.05 by ANOVA/ Bonferroni Multiple Comparison post-test.GDA^BMP^ treatment improves rotational response to amphetamine injection. Percent change of rotations per minute before and after treatment for placebo and GDA^BMP^-treatment group. Mean ± s.e.m., *n* = 5. *P < 0.05, Unpaired *t*-test.

Histological analysis of TH expression revealed that rats that had been unilaterally injected with 6-OHDA showed a > 60% reduction in levels of TH (as compared with the non-lesioned striatum) when examined at 7 weeks post lesion. In contrast, in animals transplanted with hGDAs^BMP^, the levels of TH expression were almost twice as high and achieved 70% of expression found in uninjured tissues (Fig 4A).

We assessed functional recovery as for syngeneic rat-derived cells. Three weeks after injury, animals with motor impairment were randomly assigned to two groups, a “placebo” group that received three injections of saline, and a “hGDA^BMP^ Treated” group that received three injections of 1 × 10^5^ hGDAs^BMP^ in saline. Beginning 2 days prior to transplant, all animals were immunosuppressed with cyclosporine until time of sacrifice and perfusion. Transplanted cells were detected by immunostaining for human mitochondria more than 3 weeks post-transplant.

Delayed transplants of hGDAs^BMP^ also improved behavioral performance. Rats examined 14 days after 6-OHDA injection showed significant changes in paw usage, with lesioned animals exhibiting a > 75% preference for the right paw (as contrasted with an unbiased 50% right paw preference in animals examined pre-injury; Fig 4B). Despite delaying treatment until 4 weeks after 6-OHDA injection, animals receiving hGDA^BMP^ transplants demonstrated a significant improvement in forepaw usage, and showed only a 55% right paw preference, a value statistically indistinguishable from animals examined pre-injury (Fig 4B).

Lesioned rats also showed clear evidence of amphetamine-induced rotational behavior, which was significantly reduced in rats receiving hGDA^BMP^ transplants (Fig 4C, *P* < 0.05). Comparison of amphetamine-induced rotations before and after treatment demonstrates that while the rotational behavior worsened on average by 50% in placebo group animals, all hGDA^BMP^-treated animals improved by an average of 35% over pre-treatment levels.

### GDAs^BMP^ express high levels of thrombospondin and transplantation increased synaptophysin expression in 6-OHDA-lesioned rats

Recent studies have reported that multiple CNS insults, including PD, traumatic brain injury, and Alzheimer's disease, are associated with decreased levels of synaptophysin, a pre-synaptic membrane protein expressed in neurons that has been widely used to assess synaptic density (Zhan *et al*, 1993; Eastwood *et al*, 1995). To investigate whether transplantation of GDAs^BMP^ could also affect synaptic density, we examined the expression of synaptophysin in the striata of placebo and hGDA^BMP^ treated rats.

**Figure 5:**
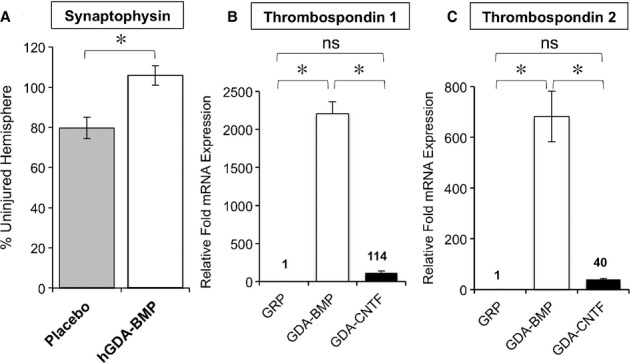
GDAs^BMP^ express high levels of thrombospondin and increase synaptophysin expression in 6-OHDA-lesioned rats. A Relative fluorescence intensity of synaptophysin staining in injured striatum of 6-OHDA injured animals after treatment with saline (placebo) or hGDAs^BMP^ shows rescue to levels seen uninjured tissue by GDA^BMP^ transplantation. Expression was normalized to synaptophysin staining in contralateral, uninjured striatum. Mean of percentage ± s.d., *n* = 3. *P < 0.05, Unpaired *t*-test. B, C Gene expression analysis of Thrombospondin 1 and 2 by semi-quantitative RT-PCR shows markedly higher levels of expression in GDAs^BMP^ than in GRP cells or GDAs^CNTF^. Average relative expression normalized to expression found in glial precursors. Mean of *n* = 3 ± s.d., *P < 0.05 for all pairwise comparisons by One-Way ANOVA and Bonferroni Multiple Comparison Test except for comparison of GRP and GDA^CNTF^ conditions (ns).

We found that in 6-OHDA lesioned rats there was a > 20% drop in levels of synaptophysin in lesioned striatum as compared with non-lesioned contralateral striatum, and that transplantation of hGDAs^BMP^ caused a rescue of synaptophysin levels to approximately 105% of the uninjured hemisphere (Fig 5A; *P* < 0.05 and Supplementary Fig 6). As expression of synaptophysin in CNS neurons has been reported to be regulated by thrombospondin-1 and 2 (Christopherson *et al*, 2005), we further examined expression of thrombospondins in GRP cells, GDAs^BMP^ and GDAs^CNTF^ and found that thrombospondin mRNA levels were significantly higher in GDAs^BMP^ than in GRP cells and GDAs^CNTF^, (Fig 5B and C). Increases were quite marked, with a > 600-fold increase in levels of thrombospondin 2 mRNA and a > 2000-fold increase in levels of thrombospondin 1 mRNA when GRP cells were induced to differentiate into GDAs^BMP^.

### Transplantation of hGDAs^BMP^ into the 6-OHDA model of PD rescues striatal populations of parvalbumin-positive interneurons

TH neurons are not the only ones lost in PD, making it of great importance to develop therapeutic approaches in which a single intervention could be used for rescuing other relevant neuronal populations. To examine this problem we focused attention on parvalbumin-expressing GABAergic interneurons. The GABAergic interneurons are essential to maintaining a balance between excitatory and inhibitory transmission in the CNS. Loss of such neurons has been observed in the thalamus of PD patients (Henderson *et al*, 2000) and in the basal ganglia and zona incerta of 6-OHDA lesioned rats (Heise & Mitrofanis, 2005; Fernandez-Suarez *et al*, 2012), resulting in an imbalance of striatal output (Salin *et al*, 2009).

**Figure 6:**
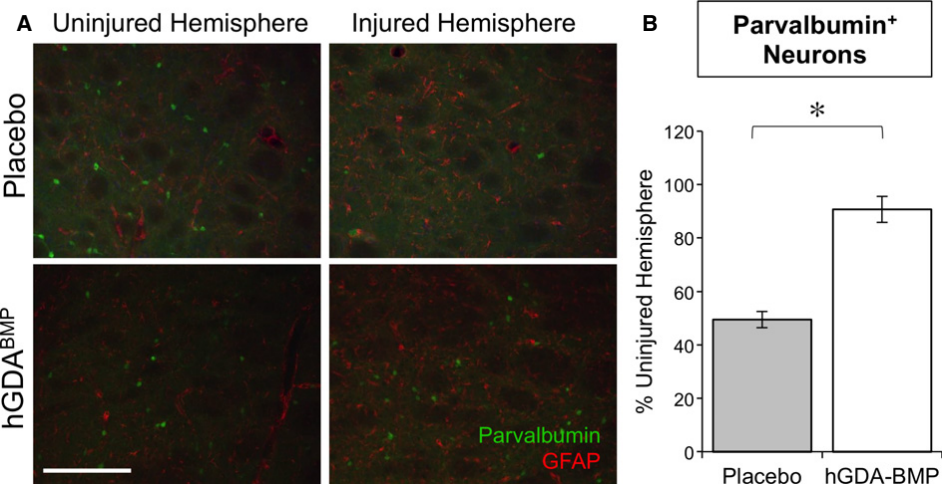
Transplantation of hGDAs^BMP^ into the 6-OHDA model of PD protects striatal parvalbumin-positive interneurons.
Parvalbumin (PV, Alexa488) and GFAP (Alexa568) staining of ventrolateral striatum in matched sections of the ipsi-and contralateral striatum of placebo and hGDA^BMP^ treated animals. PV-staining is dramatically reduced in the injured hemisphere of placebo treated animals, while hGDA^BMP^ transplantation restores the number of PV^+^ cells in the injured striatum. Scale bar = 250 μm.Quantification of PV^+^ cell numbers in the striatum. Mean ± s.e.m., *n* = 3. *P < 0.05, Unpaired *t*-test.

Delayed hGDA^BMP^ transplantation rescued parvalbumin-positive GABAergic interneuron populations in the striatum. Sections of brain were examined as for TH expression, but using antibodies against parvalbumin, glial-fibrillary acidic protein (GFAP) and DAPI (Fig 6A). Animals that had been unilaterally injected with 6-OHDA showed a 50% reduction in numbers of PV^+^ neurons when examined at 7 weeks post-lesion (as compared with the non-lesioned, contralateral striatum). In contrast, in animals transplanted with GDAs^BMP^, the number of PV^+^ cells per unit area was statistically indistinguishable from unlesioned striatum (Fig 6B).

## Discussion

Our studies demonstrate that delayed transplantation of a unique class of astrocytes, GDAs^BMP^, can simultaneously treat multiple PD-associated disease processes, above and beyond the rescue of TH^+^ neurons. Using an experimental model of PD in which GDAs^BMP^ were transplanted after the onset of motor symptoms, we found that GDA^BMP^ transplantation promoted behavioral recovery and restored levels of TH expression to control levels, despite the lack of neuronal transplantation. Transplantation of human GDAs^BMP^ also restored the numbers of parvalbumin^+^ GABAergic neurons to control levels, in what appears to be the first reported rescue of this neuronal population. Moreover, transplantation of GDAs^BMP^ also increased levels of synaptophysin, a widely used marker of synaptic density that is decreased in Lewy body diseases like PD and that has not been addressed by other approaches.

GDAs^BMP^ differ in multiple ways from previous cell types used in attempts to treat Parkinsonian pathology with support cells. For example, transplantation of cortical astrocytes isolated from postnatal animals even prior to injury into 6-OHDA lesions of the striatum provided no benefit, unless astrocytes were first genetically modified to produce GDNF (Cunningham & Su, 2002; Ericson *et al*, 2005) or to overexpress Nrf2, a regulator of antioxidant protective mechanisms (Jakel *et al*, 2007). Astrocytes isolated directly from the brain also failed to provide benefit in comparisons of olfactory ensheathing cells (OECs) and astrocytes co-grafted with ventral mesencephalic cells, in which it was found that that OECs decreased rotational behavior by an additional 10-15% over mesencephalic cells alone, while astrocytes had no effect (Johansson *et al*, 2005). In contrast, GDAs^BMP^ do not require genetic modification to provide benefit even when transplanted after the onset of disease symptoms.

Although neural stem cells (NSCs) may also provide neurotrophic support in PD-like lesions (Li *et al*, 2006; Redmond *et al*, 2007; Teng *et al*, 2011), it is not clear to what extent the support provided by NSCs is related to their ability to produce astrocytes. Such a possibility particularly needs to be considered in light of the striking differences between GDAs^BMP^ and GRP cells in their production of multiple potentially beneficial substances and their ability to provide protection *in vitro* (Figs 1 and 2), along with our previous studies demonstrating a much greater potency of GDAs^BMP^ than their parental precursor cells in providing benefit in experimental spinal cord injuries (Davies *et al*, 2006, 2008, 2011). If supportive benefits of NSC transplants are mediated by astrocytes generated from these cells, then it would seem more useful to transplant homogeneous populations of GDAs^BMP^ and eliminate any risks that might arise from inappropriate and uncontrolled differentiation of NSCs (e.g. Hofstetter *et al*, 2005). In this regard, it is also worth noting that while intrathecal delivery of BMP-7 has been shown to increase TH staining after 6-OHDA lesion (Zuch *et al*, 2004), we did not observe a direct effect of BMP4 on TH^+^ neurons *in vitro* (Supplementary Fig 2). As the neuroprotective effect of BMP4 on dopaminergic neurons previously described in mixed striatal cultures (Jordan *et al*, 1997) coincided with BMP4-induced astroglial differentiation, it is possible that the beneficial effects seen with BMP are mediated via astrocyte generation.

GDAs^BMP^ may also offer advantages over other cell types. For example mesenchymal stem cells (MSCs) suppress neuroinflammation and provide neurotrophic support in experimental models of PD (Kim *et al*, 2009; Blandini *et al*, 2010), but may still require genetic modification to boost efficacy (Lu *et al*, 2005; Somoza *et al*, 2010; Delcroix *et al*, 2011; Shi *et al*, 2011). Retinal pigment epithelial (RPE) cells also have been examined as a type of non-neuronal cell therapy due to their intrinsic production of levodopa (a potential precursor for dopamine production) (Cepeda *et al*, 2007), but failed to provide benefit in clinical trials (Gross *et al*, 2011). There is no evidence that RPE cells might rescue GABAergic neurons or synaptophysin expression.

It seems likely that one of the reasons for the efficacy of GDAs^BMP^ is the multiplicity of potentially beneficial agents they produce. In addition to our previous demonstrations that these cells produce BDNF and GDNF (Davies *et al*, 2008, 2011), we now found that GDAs^BMP^ also produce other compounds of great interest in PD therapy, including neurotrophic factors (neurturin, IGF-1 and IGF-2), modulators of synaptogenesis (THBS1, THBS2) and antioxidants such as glutathione. These properties underscore the singular nature of these cells, as these properties were not expressed by their immediate precursors (i.e. GRP cells) or (with the exception of neurturin) even by astrocytes derived from GRP cells exposed to CNTF (i.e. GDAs^CNTF^), despite being generated from the same precursor population that gives rise to GDAs^BMP^. Although we did not transplant either GRP cells or GDAs^CNTF^, their lack of benefit *in vitro* and lower levels of production of BDNF, GDNF, IGF-I, THBS1, THBS2 and GSH all suggest that, as in previous studies on experimental spinal cord injury (Davies *et al*, 2006, 2008), GRP cells and GDAs^CNTF^ are unlikely to provide the benefits achieved by transplantation of GDAs^BMP^.

One intriguing example of the potentially multiple actions of GDAs^BMP^ was provided by restoration of normal levels of expression of synaptophysin *in vivo*. When compared with their ancestral GRP cells, GDAs^BMP^ showed a > 600-fold increase in levels of THBS2 mRNA, and a > 2000-fold increase in mRNA levels for THBS1, two of the most potent molecules for promoting synaptic efficacy (Liauw *et al*, 2008) and dendritic spine formation (Garcia *et al*, 2010), processes known to be compromised in PD (Bezard, 2010; Ingham *et al*, 1989; McNeill *et al*, 1988; Nitsch & Riesenberg, 1995; Soderstrom *et al*, 2010). In addition, it also is known that making cells more reduced can increase their sensitivity to survival factors (Mayer & Noble, 1994). As astrocytes are the major source of neuronal GSH (Dringen *et al*, 2000; Wang & Cynader, 2000; Shih *et al*, 2006; Rana & Dringen, 2007), the increased production of GSH by GDAs^BMP^ may further contribute to their ability to promote recovery of distinct neuronal populations. This is not only of particular interest due to the importance of oxidative stress in PD (for examples and reviews see Gotz *et al*, 1990; Jenner, 1991; Smeyne & Smeyne, 2013; Zhou *et al*, 2008), but may also represent a novel mechanism by which the activity of multiple signal pathways may be enhanced. Finally, while there were no apparent effects of GDA^BMP^ transplantation on resident microglial cells or astrocytes as assessed by ED1 and GFAP labeling, we cannot exclude the possibility that GDA^BMP^ transplants may also act via modulation of inflammatory responses.

The restoration of levels of TH from <40% of levels seen in uninjured tissue to levels statistically indistinguishable from uninjured tissue, and the restoration of numbers of parvalbumin^+^ GABAergic interneurons from <50% of that seen in uninjured tissue to values also indistinguishable from normal tissue, raises interesting questions about the biological processes underlying these effects. Restoration of TH levels could occur for multiple reasons, such as restoration of function in neurons that were damaged but still present, promoting sprouting of existing neurons, or even recruiting of new neurons from stem cells in the adjacent subventricular zone (SVZ). For example, as it is known that BDNF administration can recruit new interneurons from precursor cells in the SVZ in experimental models of Huntington's disease (Benraiss *et al*, 2012), it is reasonable to speculate that GDAs^BMP^ might have similar effects in 6-OHDA-induced lesions. The relative importance of each of these possible contributions to the recovery observed will be the subject of future research. It will also be of interest to determine whether part of the problem in PD is that astrocyte function is itself compromised, as has been observed for amyotrophic lateral sclerosis and other neurological afflictions (Dietrich *et al*, 2005; Seifert *et al*, 2006; Nagai *et al*, 2007; García-Matas *et al*, 2008; Sidoryk-Wegrzynowicz *et al*, 2010). If this were the case, this would increase still further the importance of focusing attention on therapies capable of restoring astrocytic function.

Perhaps most importantly, GDA^BMP^ transplantation appears to offer a promising inroad into one of the most challenging problems in the development of PD therapies, which is developing therapeutic approaches to the diversity of pathological processes occurring in this disease. Many of these processes, such as neuronal survival, synapse formation, control of oxidative stress and modulation of inflammation, are normally regulated by astrocytes and the harnessing of astrocytes to correct such pathologies may therefore be of considerable value. Although improvements in behavior in our experiments were not quite as complete as those observed after direct transplantation of TH expressing neurons into 6-OHDA rat models targeting the median forebrain bundle (MFB) or substantia nigra pars compacta (both of which lesions cause a more rapid and complete loss of striatal TH^+^ cells) (e.g. Bjorklund *et al*, 2002; Ben-Hur *et al*, 2004; Cho *et al*, 2008; Wernig *et al*, 2008; Yang *et al*, 2008; Cai *et al*, 2010; Kriks *et al*, 2011), the improvements delivered by GDAs^BMP^ did not require any neuronal transplantation. Thus, while neuronal transplantation can address loss of a specific neuronal population, GDA^BMP^ transplantation is thus far the only reported approach that addresses a broader range of PD-related dysfunctions. The development of such strategies for slowing or reversing the degeneration of multiple populations of neurons in models of PD hence offers an important complement to strategies focused on dopaminergic neuron or neurotransmitter replacement by offering the opportunity of obtaining clinically important benefits without having to incorporate transplanted neurons into existing circuitry. Moreover, improving the supportive environment of the CNS may not only be of value by itself, but may also prove of importance in optimizing utility of neuronal replacement therapies as progressive neurodegeneration could compromise the efficacy of the transplanted neurons (Hansen *et al*, 2011; Kordower *et al*, 2011).

The rescue of parvalbumin-expressing GABAergic interneurons by delayed transplantation of GDAs^BMP^ is a particularly intriguing example of the potential value of such approaches. This is a population of neurons that is decreased in both PD and in 6-OHDA models of PD (Fernandez-Suarez *et al*, 2012; Southwell *et al*, 2012), and loss of inhibitory projections to the subthalamic nucleus may compound the effects of dopaminergic loss, leading ultimately to reduced excitatory output to the motor cortex and associated cortical regions (Fernandez-Suarez *et al*, 2012). No previous reports have indicated the possibility of rescue of these cells and there is no information even available on what trophic factors might be able to provide such rescue. Yet GDAs^BMP^ were also able to rescue this population, just as they rescued multiple neuronal populations in experimental spinal cord injuries (Davies *et al*, 2006, 2008, 2011).

The demonstration that the multiple gains we observed can be obtained by delayed transplantation of a population of well-defined human GDAs^BMP^, the precursors of which can be readily expanded *in vitro* to achieve upwards of several billion cells in fewer than ten passages prior to induction of differentiation, provides a candidate therapeutic agent that delivers multiple benefits with transplantation after disease symptoms are present. This outcome is very different from our previous demonstrations of the utility of GDAs^BMP^ in acute spinal cord injuries (Davies *et al*, 2006, 2008, 2011). GDAs^BMP^ could theoretically be delivered after appearance of symptoms but early enough in the disease process to forestall further degeneration and to rescue multiple neuronal populations. Moreover, the relevance of oxidative stress and the potential benefit of such factors as BDNF, GDNF and IGF-1 in multiple other neurological diseases (e.g. ALS and Huntingdon's chorea) indicate obvious potential applications of GDAs^BMP^ in other settings. The ability of these cells to support survival of multiple neuronal subtypes, promote neurite outgrowth and expression of synaptophysin, modulate reactive gliosis and combat oxidative stress demonstrates their utility as multi-purpose support cells of the CNS (see also Davies *et al*, 2006, 2008). To be able to address such a diversity of problems with a single human cell type appears to finally allow the harnessing of the apparent potential of astrocytes as a vehicle for CNS repair.

## Materials and Methods

### Isolation of glial restricted precursors and preparation of GDAs^BMP^ and GDAs^CNTF^and preparation of conditioned media

Glial precursors and glial precursor-derived astrocytes were prepared as described (Rao *et al*, 1998; Davies *et al*, 2008, 2011). Rat GRPs were isolated from the spinal cord of E13.5 F344 hPAP^tg^ embryos. Human precursors were obtained from the rostral neural tube of 9 and 10 week old elective abortus samples using the Safe-Harbor method, as approved by the Research Subject Review Board. After removal of the meninges, tissue was digested at 37°C with 59 U/ml papain (Worthington, Lakewood, NJ) in Hanks balanced salt solution (HBSS, Invitrogen, Carlsbad, CA) supplemented with 10 mM Hepes (EMD, Billerica, MA), pH8.0 and 125 U/ml DNase I (Sigma, St. Louis, MO), and triturated in 0.2%(w/v) BSA/HBSS (Sigma, St. Louis, MO), 80 KU/ml DNase I. A2B5^+^PSA-NCAM^**−**^ glial progenitor cells were isolated by step-wise immunopurification using anti-PSA-NCAM and A2B5-bound magnetic beads (Miltenyi, Bergisch Gladbach, Germany) and cultured in 5% O_2_/ 7% CO_2_ in Bottenstein-Sato F12 medium with 10 ng/ml human recombinant basic fibroblast growth factor (Peprotech, Rocky Hill, NJ) on a substrate of fibronectin (Chemicon, Temecula, CA) and laminin (Invitrogen). Upon differentiation of GDAs in Bottenstein-Sato F12 medium containing 1 ng/ml bFGF and either 10 ng/ml of BMP4 or 10 ng/ml CNTF, cultures were washed twice with one culture volume of prewarmed Neurobasal with B27 supplement without antioxidants (NB-B27 no AO, Invitrogen, Carlsbad, CA) and without BMP4 or CNTF, and then cultured for another 48 h in one volume of NB-B27 without antioxidants prior to harvest and sterile filtering of the conditioned medium.

### Neuron survival assay

Cortical and dopaminergic mid-brain neurons were isolated from E18.5 Sprague-Dawley rats and plated on poly-L-lysine coated plates in complete Neurobasal/B27 (Invitrogen). Mid-brain neurons were cultured with GDNF (10 ng/ml), BDNF (10 ng/ml) and dbcAMP (0.5 mM) for 6 days for dopaminergic neuron maturation. Cells then were switched to Neurobasal/B27 media without antioxidants, supplemented with GRP, GDA^BMP^ or GDA^CNTF^ conditioned medium (CM) for 24 h. In some groups, cells were further challenged by addition of tert-butyl hydroperoxide (tBOOH,1 μM, Sigma, St. Louis, MO) or 6-hydroxy-dopamine (6-OHDA, 10 μM, Sigma, St. Louis, MO). Cell survival was determined by co-labeling cultures with Tuj1 and anti-Tyrosine hydroxylase antibody (Secondary antibodies: anti-mouse IgG1-Alexa 488 and anti-rabbit Ig Alexa 568, Invitrogen). TujI^+^ or TujI^+^/TH^+^ neurons were counted using a Celigo® adherent cell cytometer (Brooks, Chelmsford, MA). Results are presented as mean ± s.d. (*n* = 3). Statistical analysis was performed using One-Way ANOVA, pairwise Bonferroni Multiple Comparison Test (*P < 0.05; Prism 5, Graphpad, La Jolla, CA).

### Neuroprotective agent measurements

GRPs were plated in a 6-well plate at 2000/cm^2^ density in DMEM/F12 media with N2 supplement and fibroblast growth factor (FGF, 10 ng/ml). GRPs were either maintained as precursor cells in FGF containing medium or differentiated into GDA by growth factor withdrawal and BMP or CNTF exposure for 6 days. After differentiation, mRNA expression analysis of BDNF, GDNF, IGF1, IGF2, Neurturin and thrombospondin-1 and 2 was performed by reverse-transcriptase semi-quantitative polymerase chain reaction (RT-QPCR), as previously described (Davies *et al*, 2011). Multiplex QPCR reactions of RT product were performed using FAM-labeled probes in combination with VIC-labeled, primer limited GAPDH probe and Taqman mastermix (all Applied Biosystems, Foster City, CA). △△*C*_t_ analysis was performed using Microsoft Excel software as previously described (Livak & Schmittgen, 2001). Independent experiments were performed in triplicate and average fold change in expression normalized to expression in undifferentiated GRP cells grown in bFGF. Values presented as mean values. Statistical analysis was performed using One-Way ANOVA, followed by pairwise Bonferroni Multiple Comparison Test (Prism 5, Graphpad, La Jolla, CA). *P* < 0.05 for pairwise comparisons except: GRP versus GDA^CNTF^ for GDNF, BDNF, IGF1 and GRP versus GDA^BMP^ for Neurturin). Expression of GCLC (Lab Vision, Fremont, CA) and GPx (Abcam, Cambridge, UK) were measured by Western blot analysis (Li *et al*, 2007).

### ROS measurement

GRPs or GDAs^BMP^ were plated in 96-well plate at 10 000/cm^2^ density in NB/B27-AO. After 4 h, cells were incubated in phenol red free medium containing 5 μM H_2_DCFDA (Invitrogen, Carlsbad, CA) for 20 min. ROS level was determined by the intensity of fluorescence at 488/525. Statistical analysis was performed using One-Way ANOVA, followed by pairwise Bonferroni Multiple Comparison Test (*P < 0.05).

### LC-MS/MS measurements

GSH and NADPH/NADP^+^ level were measured by LC-MS/MS as previously described (Bajad *et al*, 2006; Munger *et al*, 2006). Cell extracts were prepared by aspirating media and quickly adding 50:50 ice cold TBA solution (10 mM tributylamine, 15 mM acetic acid in 97:3 water:methanol):MeOH. The samples were incubated at −80°C for 10 min and the cell extracts were harvested into 1.5 ml tubes. Extracts were vortexed and pelleted before the resulting supernatants were analyzed by LC-MS/MS using reversed phase chromatography with an amine-based ion pairing agent coupled to an electrospray mass spectrometer running in negative mode. For HPLC (C18 150 × 2.00 mm column with a 4 μm particle size, Phenomenex, Torrance, CA), solvent A = TBA solution and solvent B = 100% methanol. The HPLC gradient was as follows: *t* = 0, 0% B; *t* = 5, 0% B; *t* = 10, 20% B; *t* = 20, 20% B; *t* = 35, 65% B; *t* = 38, 95% B; *t* = 42, 95% B, *t* = 43, 0% B; *t* = 50, 0%. LC instrumentation was an LC-20 AD HPLC system (Shimadzu, Kyoto, Japan), injection volume 20 μl. MS instrumentation was a TSQ Quantum Ultra triple-quadruple mass spectrometer (Thermo Fisher Scientific, Waltham, MA). Parameters were as per (Munger *et al*, 2006) and metabolite specific mass spectrometry parameters were as per (Bajad *et al*, 2006), with the exception of measurement of oxidized glutathione. Oxidized glutathione was measured using an MRM scan of 611 to 305 m/z at 28 eV. Oxidized glutathione pools were taken as total glutathione due to the complete oxidation of the reduced glutathione pool during sample handling, which was verified by MRM-specific scan for reduced glutathione (306 to 143 m/z at 17 eV). Resulting metabolite signal intensities were analyzed by the Xcalibar software. Signal intensities were normalized to the protein content of lysates. Statistical analysis was performed using One-Way ANOVA, pairwise Bonferroni Multiple Comparison Test (*P < 0.05).

### Injury model and transplants

Animal experiments were conducted in in accordance with the University of Rochester Medical Center animal care standards. Fisher 344 adult male rats were obtained from Charles River Laboratories. Stereotactic injection of 7 μg 6-6-OHDA (Sigma, St. Louis, MO) into the right side pars compacta of the striatum was performed at three coordinates (Bregma: ML/AP, −2.6/1.4, −3.4/0.4, −4.0/−0.8). Four weeks post injury, animals underwent a second surgery at the original injection coordinates, receiving either three injections of saline (placebo) or three injections of GDAs^BMP^ for a total of 3 × 10^5^ cells (GDA^BMP^ Treated). Animals receiving hGDAs^BMP^ were immunosuppressed with cyclosporine (10 mg/kg, LC Laboratories, Woburn, MA). Immunolabelling revealed that GDAs^BMP^ persisted in all animals, both in syngeneic rat transplants and after transplantation of human GDAs^BMP^ into immunosuppressed recipients (Supplementary Fig 4).

### Behavioral analysis

Cylinder Test/Paw preference test was performed as previously described (Schallert *et al*, 2000): The number of ipsilateral (right) paw contacts was calculated as a percentage of total paw touches, and expressed as average ± s.e.m. Rotational behavior was measured following injection of 5 mg/kg D-methamphetamine (Kelly *et al*, 1975). Complete rotations were scored and mean rotations per minute calculated. Based on rotations per minute, the percent change between pre-treatment and post-treatment performance was determined for each animal, and the average and s.e.m. of each treatment group calculated. Statistical analysis was performed using One-Way ANOVA, followed by pairwise Bonferroni Multiple Comparison Test (*P < 0.05).

### Immunofluorescence

Histological samples were prepared as previously described (Davies *et al*, 2011). Three weeks post-injury we perfused animals with 4 units/ml of heparin sulfate and 4% paraformaldehyde in phosphate-buffered saline (PBS), removed the whole brains and cryoprotected the tissue in 30% w/v sucrose. We collected 40 μm frozen sections from each using a sliding microtome and stored these at 4°C in phosphate buffer with 0.03% NaN_3_. Immunofluorescence was used to examine every sixth section of the striatum over a span of approximately 1.2 mm from the rostral corpus collosum to the rostral hippocampus. Floating sections were permeabilized in 0.3% Triton X-100 and probed overnight at 4°C with the following antibodies, all diluted in 0.1 M phosphate buffered saline plus 10% normal goat serum with 0.3% Triton x-100: anti-synaptophysin (Sigma, St. Louis, MO), anti-TH (Millipore), anti-ED1 (Abd Serotec, Oxford, UK), anti-GFAP (Dako, Glostrup, Denmark), anti-parvalbumin (Chemicon). Secondary antibodies were conjugated to Alexa-488 or 568 (Invitrogen). Nuclei were stained with DAPI (4′,6-diamidino-2-phenylindole, dihydrochloride; Invitrogen). Sections were rinsed with phosphate buffer, dried and mounted with fluoromount-G (SouthernBiotech, Birmingham, AL) and coverslipped with glass. For quantification of tyrosine hydroxylase and synaptophysin expression, tissue was imaged on an upright fluorescent 80i Nikon microscope and analyzed using NIS Elements® software for total fluorescent intensity. The dorso-medial region of the striatum was analyzed, *n* = 3 sections per animal, three animals per cohort. For counts of parvalbumin expressing cells, tissue was imaged on a Leica Laser Confocal microscope and analyzed using LAS AF® software. The entire area of the striatum was analyzed, *n* = 3 sections per animal, three animals per cohort.

**The paper explained**

**Problem**

Parkinson's disease is a progressive neurodegenerative disease that affects multiple neuron populations. Moreover, neurons that are still present may be impaired and functionally unable to compensate for lost function. The predominant current cellular therapies, aimed at replacing dopaminergic neurons, do not treat the full range of pathological processes relevant to a comprehensive treatment. In addition, therapies focused on the delivery of single trophic factors, or pharmaceutical interventions that target only dopaminergic neurons, do not address the broader problems pertinent to Parkinsonian pathology. Thus, there is a great need to identify therapeutic approaches that target multiple problems that occur in the Parkinsonian central nervous system to be addressed, preferably with a single multi-potent therapy.

**Results**

Post-symptomatic transplantation of GDAs^BMP^, a unique class of glial precursor-derived astrocytes, into a neurotoxic model of PD results in functional improvement and recovery of at least two distinct neuronal populations in the striatum, dopaminergic neurons and parvalbumin-expressing GABAergic interneurons. GDAs^BMP^ intrinsically express a cocktail of factors, several of which have been shown to support neuron survival in models of PD. These include glial-derived neurotrophic factor, brain-derived neurotrophic factor, insulin-like growth factor I and neurturin. In addition GDAs^BMP^ secrete the regeneration-and synaptogenesis-promoting factors thrombospondin 1 and 2, and also produce increased amounts of glutathione, one of the major protectors against oxidative damage. As predicted from this group of substances, GDAs^BMP^ support neuronal survival, protect from oxidative stress and increase expression of synaptic proteins in the lesioned striatum. Thus, GDAs^BMP^ not only support survival of different neuronal populations but also may improve neuron functionality.

**Impact**

GDA^BMP^ transplantation is the first example of a multimodal support cell therapy with the potential to promote recovery of multiple neuron populations of relevance to PD with a single therapeutic approach, and provides the first approach that rescues both the dopaminergic and parvalbumin-expressing GABAergic neurons that are lost in PD. The availability of human GDAs^BMP^, which can be readily produced in therapeutically relevant amounts, also provides the first opportunity to harness the beneficial properties of astrocytes in treatment of CNS injury. Consequently, GDA^BMP^ therapy may provide a novel treatment for PD that addresses multiple pathologies relevant to this disease, but also are likely to be equally useful for treatment of other neurodegenerative diseases.
