# Supplementary material for: Delayed transplantation of precursor cell-derived astrocytes provides multiple benefits in a rat model of Parkinsons
Source: EMBO Mol Med. 2014 Jan 29;6(4):504–18. doi: 10.1002/emmm.201302878 (PMC3992077; doi:10.1002/emmm.201302878)
Supplement: Supplementary file 1 [file emmm0006-0504-sd1.pdf]

EMBO Molecular Medicine

Delayed transplantation of precursor cell-derived astrocytes provides multiple benefits in a rat model of Parkinsons.

Christoph Proschel, Jennifer L. Stripay, Chung-Hsuan Shih, Joshua C. Munger, Mark D. Noble

Corresponding author: Christoph Proschel, University of Rochester

Review timeline:

Submission date:	11 April 2013
Editorial Decision:	03 May 2013
Revision received:	25 October 2013
Editorial Decision:	12 November 2013
Additional Author Correspondence	27 November 2013
Additional Editorial Correspondence	28 November 2013
Revision received:	13 December 2013
Accepted:	16 December 2013

Transaction Report:

(Note: With the exception of the correction of typographical or spelling errors that could be a source of ambiguity, letters and reports are not edited. The original formatting of letters and referee reports may not be reflected in this compilation.)

Editor: Céline Carret

1st Editorial Decision 03 May 2013

Thank you for the submission of your manuscript to EMBO Molecular Medicine. We have now heard back from the three referees whom we asked to evaluate your manuscript. Although the referees find the study interesting, they also raise a number of concerns that should be addressed in a major revision of this work.

As you will see from the reports below, while referees 1 and 3 are positive about the study, referee 2 is much more concerned by the limited mechanistic insight. Overall, clarification of the findings, and provision of better and more appropriate controls are required to improve the impact of the study and will help to get at a mechanism.

In our view the suggested revisions would render the manuscript much more compelling and interesting to a broad readership. We therefore hope that you will be prepared to undertake the recommended experimental revision.

Please note that it is our journal's policy to allow only a single round of revision, and that acceptance or rejection of the manuscript will therefore depend on the completeness of your response and the satisfaction of the referees with it.

EMBO Molecular Medicine has a "scooping protection" policy, whereby similar findings that are published by others during review or revision are not a criterion for rejection. Should you decide to submit a revised version, I do ask that you get in touch after three months if you have not completed it, to update us on the status.

Please also contact us as soon as possible if similar work is published elsewhere. If other work is

published we may not be able to extend the revision period beyond three months.
I look forward to seeing a revised form of your manuscript as soon as possible.

***** Reviewer's comments *****

Referee #1:

This is an exciting study which demonstrates that astrocytes derived from GRPs in the presence of BMP are an important candidate for repair strategies for PD. This is a well executed study which coveres molecular, in vitro and in vivo studies demonstrating the potential advantage of astrocytes derived from GRP using BMP. The authors have used a good animal model for PD and demonstrated significant behavioural and biochemical results. My only comment would to request some representative images of the immunoreactivity reported in grafts and comments on other cellular effects that could occur as a consequence of the transplantation of GRP cells.

Specific points:
1) A little more information about the CNTF treated GRPs in the introduction would help the reader get the results meaning quicker. The data was presented about the two various means to provide astrocytes from GRPBMP but the reader was not lead easily into the concept that the astrocyte are different. It is clearer later on in the manuscript though.
2) Please show representative images of the control and experimental TH, synaptophysin and Thrombospondin 1 and 2 immunoreactivity.
3) Could the authors comment on how the endogenous astrocytea react to cell transplantation? Is there a change in their reactivity. In addition, is there any change in the phenotype of the endogenous OPCs? This is because an increase in BDNF, IGFs may well impinge on endogenous glial cells.
4) Linked to 3) is there any reason why secreted growth factors from the GFPCNTF cells may have a negative influence on endogenous neural cells?
5) Lastly how are microglial cells affected by the graft?

Referee #2:

The current paper by Proschel et al., utilizes a class of astrocytes generated from glial-restricted precursor (GRP) cells that have been differentiated with either BMP or CNTF. They show, by qPCR, that the BMP differentiated cells have higher levels of GDNF, BDNF, IGF1 and 2 and neurturin, molecules previously shown to have beneficial effects following 6-OHDA lesions. In addition, the authors show GRP cells treated with BMP have higher levels of proteins associated with oxidative stress. They then treat primary midbrain dopaminergic neurons with the conditioned media (CM) from the BMP, CNTF or undifferentiated GRP cells. The CM from the BMP treated cells, but not CNTF treated cells, was able to promote survival of TH+ cells. Further, the authors show CM from the BMP treated cells promoted the survival of dissociated cortical neurons and cortical neurons treated with a pro-oxidant, consistent with the CM from the BMP treated cells containing factors that promote neuronal survival.

Given the benefits seen in the dissociated neurons, the authors then transplanted the GDPBMP cells into rats that had previously been given unilateral injections of 6-OHDA into the pars compacta of the striatum. Seven weeks following transplant, TH expression in the injured striatum was shown to be similar to the unlesioned striatum, while the saline treated animals showed a 70% reduction in TH levels. Similar to the rat derived GDA cells, the authors then generated human GDA cells treated with BMP4 and showed the human cells were able to promote recovery, although not to the extent the rat cells did.

The authors next tested behavioral outcomes following the lesion and transplantation of the GDABMP cells. Rats examined fourteen days following 6-OHDA lesions were monitored for motor impairments, and D-metamphetamine-induced rotational behavior. Animals showing defects were assigned into one of two groups, one given an injection of saline the other transplanted with GDABMP cells. Following transplantation the animals receiving the BMP stimulated cells showed

recovery of paw reaching and D-metamphetamine rotational behavior. Similarly, rats transplanted with GDABMP cells derived from humans showed positive histological and behavioral effects following 6-OHDA treatments. The authors have also shown that treatment with the GDABMP cells leads to increases in synpatophysin staining and the number of parvalbumin positive cells in the treated striatum.

Major Points.

Overall this was an interesting study; however, the overall impact of the results seem limited in scope given the large number of studies showing that TH+ staining, and behavioral deficits, can be improved with the administration of various growth factors following 6-OHDA. In the current study BMP treatment has been found to increase the levels of GDNF and BDNF as measured by qPCR. Whether the levels of protein are sufficient to account for the effects was not tested. My lack of enthusiasm for the current study stems from the fact that the current study is very similar to other studies published by this group. This group has previously shown that the GDABMP cells, when transplanted into the spinal cord following injury, can induce axon outgrowth/survival, while the GDACNTF cells or GRP cells fail to show beneficial effects. Thus, the current study uses a similar paradigm to studies from this group - only here they have adapted it to a PD model. Overall this doesn't seem like a major advance given the impact and scope of the journal.

I would also suggest that a number of controls are necessary to strengthen the conclusions the authors have suggested. For example, in the dissociated neuron study, it is important to control for the addition of BMP4 alone. Since BMPs have been added to the GRP cells to induce differentiate, BMP4 is still likely present in the conditioned media. Since the CM was used to show an effect on TH+ve cells, how can they rule out that residual BMP4 did not have any effects? This is important given the BMPs have been shown to support the survival of dopaminergic neurons in the past. Similarly, is CNTF alone having a negative effect on cell survival? Ideally, functional blocking antibodies for BMP4 or CNTF need to be added to the CM prior to its addition to the dissociated neurons.

In the current study only the GDABMP cells were transplanted and compared to saline injections. Why weren't the GDACNTF cells or the undifferentiated RGD transplanted following 6-OHDA treatments? The results would be more convincing if these controls were utilized.

All histological counts were done on an n=3 with only 3 sections counted/animal. Given the section thickness was only 40um and the thickness of the striatum is significant, I question how relevant counting 3 sections/animal is. For the parvalbumin counts it does not seem proper stereology was used to allow for the same segmental region being counted. This may significantly affect the numbers.

It is unclear to me when the behavioral tests were done following 6-OHDA lesions, and how this timeline is related to the histological studies. It seems that the authors are testing the behavior 1 week following cell transplantation. They state in the paper "Rats examined at fourteen days post 6-OHDA injection showed significant changes in paw usage........In marked contrast, animals that received GDABMP transplants 3 weeks after 6-OHDA injection demonstrated a significant improvement in forepaw usage." I understand this to mean animals were tested 'fourteen days' (or two weeks) after 6-OHDA treatments to assign them either a placebo or treatment group, (ie. they were given saline or GDABMP cells). The animals were then tested "3 weeks after 6-OHDA injections" or one week after the injection of either saline or cells. Thus the animals showed functional recovery 1 week following treatment. This seems to be different than the timeline in the supplemental figure. Since most of the anatomy and morphology was done 7 weeks following transplant how does the functional recovery after 1 week of transplant correlate with the anatomy studies? These seem like vastly different time lines and perhaps not the same mechanism.

Referee #3:

Delayed transplantation of precursor cell-derived astrocytes provides multiple benefits in a rat model of Parkinsons.

Abstract reproduced from text
In addition to dopaminergic neuron loss, it is clear that Parkinson disease includes other pathological changes, such as loss of additional neuronal populations. As a means of addressing multiple changes with a single therapeutic approach, we have investigated a unique class of astrocytes, GDAsBMP, that are generated in vitro by directed differentiation of glial precursors. GDAsBMP produce multiple agents of
interest as treatments for PD and other neurodegenerative disorders, including BDNF, GDNF, neurturin and IGF1. GDAsBMP also exhibit increased levels of antioxidant pathway components, including levels of NADPH and glutathione, compared with other astrocytes. Besides rescuing dopaminergic neurons in
vitro from 6-hydroxydopamine (6-OHDA) toxicity, delayed transplantation of GDAsBMP into the 6- OHDA lesioned rat striatum restored tyrosine hydroxylase expression, increased numbers of parvalbumin+ GABAergic interneurons and promoted behavioral recovery. GDAsBMP expressed elevated levels of the synaptic modulatory proteins thrombospondin-1 and 2 and GDABMP transplantation restored expression of the synaptic protein synaptophysin in 6-OHDA-lesioned striata. Thus, human GDAsBMP offer a multimodal support cell therapy that provides multiple benefits without requiring prior genetic manipulation.

In this manuscript the authors suggest that a novel astrocyte population that is derived from a glial precursor by culturing in a medium containing BMP has specific protective effects in two models of PD. The authors attribute this effect to the release of trophic molecules and antioxidants by this astrocyte population.

The authors show that this effect is specific to BMP derived cells as opposed to those from the same population derived with CNTF and that the overall improvement is better than that reported in the literature with unmodified astrocytes

Comments:

1: I note that CNTF derived astrocytes make much more neurturin than BMP derived astrocytes. Do the authors fell that neurturin is not important. Likewise both cell types make relatively low levels of BDNF. Do the authors feel that BDNF is irrelevant?

2: An important concern with any astrocyte therapy or with any cell based therapy where cells are providing trophic support is to consider levels in vivo. A variety of literature shows that astrocytes in response to neuronal injury will respond with trophic factor release. Adding a modest number of additional cells that release additional levels of the same trophic factors is unlikely to have much benefit and indeed delivery of large amounts of such factors has had modest effects at best.

3: An important experiment that needs to be done is to show persistence of astrocytes that were transplanted in the lesion site. Given human cells were transplanted this should be possible. MSC are not persistent and generally have transient effects and much of their effects have been related to modulation of theimmune system and reduction in inflammation. It may be useful to examine if these astrocytes modulate inflammation

4; The delayed addition is a welcome strategy but I find the positive result somewhat surprising. IT suggests that TH is downregulated rather than the cells are lost to show such recovery and innervation. If the authors believe otherwise they should say so and and if not they should discuss possibilities. I note their culture model should allow them to test if the conditioned medium upregulates TH expression.

5: Likewise the culture experiments should be better described. One presumes that the cultures were not pure cultures but contained a mixture of astrocytes endothelial cells, microgla etc. If this is the case the others should clarify as the effect of the astrocytes could be indirect.

6; In general when one has a nice system and sees an effect with conditioned medium one would like to see if one can block the effect of the candidate molecules or at least attempt to do so.

© EMBO

7: The discussion is well written and the results carefully interpreted but I note one important comparison that is missing and that is transplant of dopaminergic neurons themselves. The rotational behavior improvement, the recovery and the effectiveness do delayed transplants is generally better at least as reported in the literature. The authors should perhaps add that to their discussion along with a discussion of availability of their cells in numbers sufficient to move to translation.

Overall I think this is a well written manuscript that has performed good controls to make a case for this class of astrocyte population to be a legitimate candidate for a cell based therapy. However, I would like to see some of the concerns I have raised in the comments addressed.

1st Revision - authors' response 25 October 2013

We thank the reviewers for their positive comments and for constructive and thoughtful criticisms. The reviewers agreed that this is an interesting study and the majority felt this was a well executed study using an appropriate animal model and good controls, "making a case for this class of astrocyte population (GDAs^{BMP}) to be a legitimate candidate for a cell based therapy".

We provide here a detailed response to the reviewers. In order to comply with journal space limits, some information requested has been added to supplemental information. In addition, the discussion as a whole has been restructured to better address the reviewers' comments, and changes referring to reviewers' questions are highlighted in italic font.

Referee #1:

"1) A little more information about the CNTF treated GRPs in the introduction would help the reader get the results meaning quicker. The data was presented about the two various means to provide astrocytes from GRPBMP but the reader was not lead easily into the concept that the astrocyte are different. It is clearer later on in the manuscript though."

We have added additional information to the introduction, to introduce the CNTF induced population of astrocytes (GDAs^{CNTF}), and highlighting some of the major known differences with the BMP induced astrocytes.

"2) Please show representative images of the control and experimental TH, synaptophysin and Thrombospondin 1 and 2."

Representative images of brain sections labelled with tyrosine hydroxylase or synaptophysin antibody have been added to the supplemental information in figure 5 and figure 6. As both thrombospondin 1 and 2 are secreted proteins, immunofluorescence labelling of sections was uninformative beyond the fact that thrombospondin 1 and 2 are expressed by GDAs^{BMP}.

It is important to note that the inability to visualize thrombospondin in vivo could be for many reasons, none of which counter the core hypothesis and observations of this manuscript. Diffusion of thrombospondin from the site of cell injection, coupled with secretion by the GDAs^{BMP} would reduce visualization without altering the observations that the astrocytes secrete these proteins and, most critically, that the increased levels of synaptophysin predicted by thrombospondin production were also observed.

"3) Could the authors comment on how the endogenous astrocytes react to cell transplantation? Is there a change in their reactivity (GFAP). "

At the time points assessed, both saline treated and GDA^{BMP} transplanted animals demonstrated only slightly increased GFAP labelling of resident astrocytes in the lesioned striatum. We have inserted text commenting on this in paragraph 4 of the discussion.

"In addition, is there any change in the phenotype of the endogenous OPCs. This is because an increase in BDNF, IGFs may well impinge on endogenous glial cells."

We were puzzled by this request in the context of the present manuscript, even though it is a question we think may be relevant to understanding the effects of $GDAs^{BMP}$ in other types of lesions. In the context of Parkinsonian lesions, however, a potential contribution of cells of the oligodendrocyte lineage to damage or recovery is not something that has been the subject of much attention.

Although one can speculate in interesting ways on potential benefits of neurotrophic factors produced by OPCs or oligodendrocytes, studies on the internal capsule (the major site of myelination in the striatum) do not appear to be part of the general discussion on important changes occurring in Parkinson's disease.

Thus, studies on OPCs would lead one – for this particular disease – into a very speculative realm. Pursuit of this question in the context of spinal cord injury, in contrast, would be very appropriate and important.

"4) Linked to 3) is there any reason why secreted growth factors from the GDACNTF cells may have a negative influence on endogenous neural cells?"

This is an interesting question. Our in vitro studies failed to reveal positive effects of $GDAs^{CNTF}$ on promoting neurite outgrowth or survival, but neither were the outcomes decreased from control levels. In the previous spinal cord transplants we carried out, it was the case that $GDAs^{CNTF}$ promoted expansion of CGRP+ fibers, and could have contributed to the pain syndromes observed in rats receiving such transplants. We are not aware of analogous neuronal populations that might be studied in the context of Parkinsonian lesions. Moreover, the transplantation of $GDAs^{CNTF}$ into experimental models of spinal cord injury did not have any beneficial effect on axonal regeneration.

One can speculate that $GDAs^{CNTF}$ could have other deleterious effects, but their inability to even provide benefit in vitro makes their study in vivo a topic of quite limited interest in models of Parkinson's disease at this time. We hope this might change, as we think the biology of $GDAs^{CNTF}$ does need to be further explored. But any outcome from such studies would not alter the data regarding the positive effects of transplanting $GDAs^{BMP}$.

"5) Lastly how are microglial cells affected by the graft?"

We agree that this would be very interesting to know, but so far we don't see anything of note. ED1 labelling of microglia revealed no significant differences between saline and GDA^{BMP} treated animals. In both cohorts the number of ED 1 labelled monocytes and microglia remained elevated. We cannot exclude the possibility that GDA^{BMP} transplants may also act via modulation of inflammatory responses that are controlled by microglia, but currently that would be a purely speculative idea.

Referee #2:

We will take the comments of Referee 2 slightly out of order so that we can clear up an apparent point of failed communication immediately.

"My lack of enthusiasm for the current study stems from the fact that the current study is very similar to other studies published by this group. This group has previously shown that the GDABMP cells, when transplanted into the spinal cord following injury, can induce axon outgrowth/survival, while the GDACNTF cells or GRP cells fail to show beneficial effects. Thus, the current study uses a similar paradigm to studies from this group - only here they have adapted it to a PD model."

This comment was dismaying to read as it indicated that we had somehow failed to communicate the multiple novel findings in the present paper. These include the following;

1. Critically, this paper is on recovery of function by transplantation of cells well *after* injury and development of motor symptoms. Our previous work on SCI was focused on discovery of a novel astrocyte population, of the importance of pre-differentiation, and of the first functional analysis by transplantation of what we hypothesized to be functionally distinct astrocyte populations. However, in the spinal cord injury model, all transplants were carried out at the time of injury (i.e., in an acute setting).

   In contrast, our present studies used delayed transplantation to restore function - a very different type of setting. Moreover, these studies were focused on testing whether the transplantation of $GDAs^{BMP}$ can be used to address problems that have not been solved by other approaches.
2. We found that delayed transplantation of $GDAs^{BMP}$ restored normal levels of a key neuronal enzyme (tyrosine hydroxylase), a finding that has no parallel in our work on spinal cord injury, and a result which was not necessarily predictable, as we are transplanting astrocytes and not dopaminergic neurons.
3. We showed delayed transplantation of $GDAs^{BMP}$ restored to normal the numbers of parvalbumin-expressing neurons, a finding that has no parallel in our previous work. Moreover, as far as we can tell (and again unlike our previous work), this is a neuronal population that no one else has previously rescued.
4. We further showed that $GDAs^{BMP}$ make multiple factors of interest beyond our previous findings on GDNF and BDNF, rescue neurons from oxidative stress and that transplantation enhances expression of the well-studied synaptic marker synaptophysin. No such work was in our previous studies.

Thus, we confess to being quite baffled by this statement and hope that we have been able to clarify the novelty and significance of our findings in the overhaul of the discussion.

"Overall this was an interesting study; however, the overall impact of the results seem limited in scope given the large number of studies showing that TH+ staining, and behavioural deficits, can be improved with the administration of various growth factors following 6-OHDA. In the current study BMP treatment has been found to increase the levels of GDNF and BDNF as measured by qPCR. Whether the levels of protein are sufficient to account for the effects was not tested."

We thank the reviewer for the kind view that this was an interesting study. It is absolutely correct that treatment with various individual growth factors has been shown to promote recovery of TH staining and behavioural deficits. At the same time, all of the factors that have been tested in Parkinson's disease clinical trials have failed to provide significant benefit. This is precisely why we feel that administration of a multimodal, cellular therapeutic may provide a means of overcoming the limitations inherent to these more pharmacological approaches. This is supported

by our finding that GDA^{BMP} transplants not only promoted recovery of TH positive neurons, but also rescued parvalbumin positive GABAergic interneurons, the first report of its kind (and a cell population for which the most relevant neurotrophic factor(s) is/are not even known.

As GDAs^{BMP} secrete a number of neurotrophic and neuroprotective factors, it is too early to say which of these proteins are responsible for the distinct benefits observed, nor would any specific outcome alter our fundamental observations.

The question of whether the levels of proteins are sufficient to account for the effects is an interesting question, but one that is very difficult to address in an accurate manner for at least two reasons. First, if we just consider the potential effects of glutathione production and effects of redox regulation on response to trophic factors, discoveries we published in PNAS in 1994 demonstrate that one cannot make any useful inferences regarding effective quantities of trophic factors. What we reported in these studies was that the amount of cell survival promoted by exposure to levels of NGF (for dorsal root ganglion neurons) and IGF-I or CNTF (for oligodendrocytes) was markedly modulated by redox state. Levels of these proteins that had little-to-no effect on survival became very effective just by co-exposing cells to enough of a cysteine pro-drug to raise their intracellular glutathione levels by 15%. The up regulation of glutathione production by GDAs^{BMP} would thus be predicted to alter these dose response curves so that lower doses of agents are effective. The second point to consider is that trophic factors (as well as mitogens) alter the response to other growth factors. For example, if you prepare oligodendrocyte progenitor cells in particular ways (as in Barres et al, 1994) you see no response to their basal mitogen (platelet-derived growth factor) unless you also add neurotrophin-3. Thus, when we have a cell that produces glutathione and multiple potentially beneficial factors, invoking the biology of standard dose-response analyses is very problematic.

It is also critical to emphasize that, as the reviewer notes, the proteins to which we refer all have been shown to be of interest on their own. The ability of GDAs^{BMP} to produce a variety of such factors (and without the need for genetic modification) is what suggested they would be of value as neuronal support systems in our experiments. Whether any particular beneficial products are necessary, or are sufficient, would not alter the outcomes of our transplantation experiments.

Barres BA, Lazar MA, Raff MC (1994) A novel role for thyroid hormone, glucocorticoids and retinoic acid in timing oligodendrocyte development. *Development* 120: 1097-1108

"I would also suggest that a number of controls are necessary to strengthen the conclusions the authors have suggested. For example, in the dissociated neuron study, it is important to control for the addition of BMP4 alone. Since BMPs have been added to the GRP cells to induce differentiate, BMP4 is still likely present in the conditioned media. Since the CM was used to show an effect on TH+ve cells, how can they rule out that residual BMP4 did not have any effects? This is important given the BMPs have been shown to support the survival of dopaminergic neurons in the past."

We thank the reviewer for reminding us to cite previous work by the Krieglstein lab (Jordan et al, 1997), in which the authors elegantly demonstrate that BMPs can promote the survival of dopaminergic neurons, and which the authors concluded was an indirect effect mediated by BMP-induced generation of astrocytes (this has been added to the discussion). More recently, the Bickford lab (Zuch et al, 2004) showed that infusion in BMP-7 into striatal 6-OHDA lesions 6 days after lesion production increased TH immunoreactivity, but no experiments were conducted to determine if this was due to direct or indirect effects of BMP. These studies have been included in the discussion section. While there are several papers on the effects of BMP on generation of neurons from CNS stem cells, we cannot find any papers showing that BMP4 directly promotes survival of dopaminergic neurons.

In respect to whether or not residual BMP could be responsible for the pro-survival effects of GDAs^{BMP}, this seems highly unlikely as our protocol for the production of conditioned medium is designed to prevent the carry-over of BMP (or CNTF) to the conditioned medium. Upon differentiation of GDAs^{BMP} in 10ml of medium with 10ng /ml of BMP4, GDA^{BMP} cultures were washed twice with 10ml of prewarmed Neurobasal with B27 supplement (without antioxidants) and without BMP4, and then cultured for another 48 hours in 10ml NB-B27 without antioxidants prior to harvest of the sterile filtered conditioned medium. Based on the surface to volume ratio of the 75cm^2 culture vessels used for production of the conditioned medium, residual BMP4 would not exceed a concentration of 0.05ng/ml, a concentration well below the EC50 of 2ng/ml described by Jordan et al (1997), and below the activity threshold for BMP4 in any assays known to us. Moreover, genome wide expression analysis suggests that differentiated GDAs^{BMP} do not produce BMP4.

Nonetheless, we directly tested the effect of 1ng/ml and 10ng/ml of BMP4 on the survival of dopaminergic neurons, and found that BMP4 did not rescue in the presence of 10µM 6-OHDA (Figure included in SI Figure 2). Two reasons may explain the difference between our results and those obtained in the study by the Krieglstein lab: first, GFAP staining of striatal cultures reveals only 1.1% (± 0.35%, StDev) of the cells are astrocytes. Second, while our in vitro essay lasts 2 days, Jordan *et al* treated cells for 8 days, which may allow sufficient time for generation of more astrocytes.

Jordan J, Bottner M, Schluesener HJ, Unsicker K, Krieglstein K (1997) Bone morphogenetic proteins: neurotrophic roles for midbrain dopaminergic neurons and implications of astroglial cells. *Eur J Neurosci* 9: 1699-1709

Zuch CL, David D, Ujhelyi L, Hudson JL, Gerhardt GA, Kaplan PL, Bickford PC (2004) Beneficial effects of intraventricularly administered BMP-7 following a striatal 6-hydroxydopamine lesion. *Brain Res* 1010: 10-16

"In the current study only the GDABMP cells were transplanted and compared to saline injections. Why weren't the GDACNTF cells or the undifferentiated RGD transplanted following 6-OHDA treatments? The results would be more convincing if these controls were utilized."

We are again concerned that we failed to communicate the goals of our studies. The studies presented in this manuscript are focused on the beneficial effects that can be obtained by transplantation of GDAs^{BMP}, as our previous studies in spinal cord injury had found that neither GRP nor GDACNTF transplants provided benefits. In addition, GDAs^{CNTF} actually resembled reactive astrocytes already present at lesion sites. Examination of the cytokine and glutathione production profiles from these cells and their lack of beneficial effects on neurons in vitro, combined with the lack of effect of these cells in studies on spinal cord injury, provides no compelling argument for transplanting these cells.

"All histological counts were done on an n=3 with only 3 sections counted/animal. Given the section thickness was only 40um and the thickness of the striatum is significant, I question how relevant counting 3 sections/animal is. For the parvalbumin counts it does not seem proper stereology was used to allow for the same segmental region being counted This may significantly affect the numbers."

Based on anatomical markers, free floating sections were carefully selected at 1.6mm AP, ~0.75mm AP and -0.4mm AP to represent as much of the area of interest as possible. (See attached figure). The injection sites for 6-OHDA and subsequent transplant sites occurred at 1.4mm AP, 0.4mm AP, -0.8mm AP). Our intent was to sample the full span of the affected area. Because of the distinct tissue morphology of the caudate putamen region in the rat brain, anatomical markers (corpus

callosum, lateral wall of the CP, etc) were sufficient to determine appropriate boundaries. We were interested in the entire dorso-lateral x ventral-medial area of the striatal region in each section and, as such, counted Parvalbumin-positive cells accordingly.

It is unclear to me when the behavioural tests were done following 6-OHDA lesions, and how this timeline is related to the histological studies. It seems that the authors are testing the behaviour 1 week following cell transplantation. They state in the paper "Rats examined at fourteen days post 6-OHDA injection showed significant changes in paw usage........In marked contrast, animals that received GDABMP transplants 3 weeks after 6-OHDA injection demonstrated a significant improvement in forepaw usage." I understand this to mean animals were tested 'fourteen days' (or two weeks) after 6-OHDA treatments to assign them either a placebo or treatment group, (i.e. they were given saline or GDABMP cells). The animals were then tested "3 weeks after 6-OHDA injections" or one week after the injection of either saline or cells. Thus the animals showed functional recovery 1 week following treatment. This seems to be different than the timeline in the supplemental figure. Since most of the anatomy and morphology was done 7 weeks following transplant how does the functional recovery after 1 week of transplant correlate with the anatomy studies? These seem like vastly different time lines and perhaps not the same mechanism.

We thank the reviewer for bringing this discrepancy to our attention and we apologize for the confusing description. The experimental plan depicted in the supplementary figure reflects the correct time points and treatment times. Four weeks after receiving unilateral 6-OHDA injections, one cohort of animals received saline injections, while the other cohort received GDA^{BMP} transplants. Behavioural testing was performed at three different times: prior to receiving 6-OHDA injections (Pre-injury), two weeks after 6-OHDA lesion and 2 weeks after transplant/saline injection. Histology was performed at three weeks post transplant (one week after completion of behaviour testing). We have corrected the text to clarify the description.

<u>Referee #3:</u>

"1) I note that CNTF derived astrocytes make much more neurturin than BMP derived astrocytes. Do the authors fell that neurturin is not important. Likewise both cell types make relatively low levels of BDNF. Do the authors feel that BDNF is irrelevant?"

We also thought this expression of neurturin was interesting and were therefore quite surprised at the lack of benefit of these cells in vitro. As discussed in the response to Referee 2, it may be inappropriate to read too much into levels of individual proteins if they are delivered in combination with increased glutathione (which was low in the $GDAs^{CNTF}$) and/or other trophic factors.

Rather than focusing on the potential importance of any one factor, the expression profile of $GDAs^{BMP}$ indicates these cells appear to provide a multifaceted collection of supportive factors that distinguishes these astrocytes from other cells that may only express one or two relevant factors (like $GDAs^{CNTF}$), or the administration of single pharmacological agents.

Nonetheless, while we did not see a significant protective effect of GDA^{CNTF} conditioned medium in our in vitro experiments it is possible that under the right conditions, neurturin expressed by $GDAs^{CNTF}$ may play a greater role.

What is clear, however, is that $GDAs^{BMP}$ express higher levels and a more complex array of neurotrophic factors than $GDAs^{CNTF}$, even though both astrocyte populations are derived from the

same precursor cells.

"2) An important concern with any astrocyte therapy or with any cell based therapy where cells are providing trophic support is to consider levels in vivo. A variety of literature shows that astrocytes in response to neuronal injury will respond with trophic factor release. Adding a modest number of additional cells that release additional levels of the same trophic factors is unlikely to have much benefit and indeed delivery of large amounts of such factors has had modest effects at best."

It is absolutely correct that this is an important concern with any cell-based therapy, which is why in vitro results can only suggest the potential value of conducting in vivo experiments. What is clear, however, is that transplantation of $GDAs^{BMP}$ had striking positive effects. Whatever the levels of individual factors that might be produced in vivo, no experimental outcome would alter the observations reported in our studies. Moreover, the delivery of cells that can secrete a battery of factors (possibly even in response to the environment) is very different, conceptually and apparently functionally from the delivery of individual trophic factors – even in large amounts.

Unlike cell replacement therapy, support cells cannot directly replace the lost neurons or myelinating cells. Instead, support cells are generally thought to promote regeneration by enabling survival of injured cells through trophic support or by inducing host precursors to replace lost cells, again through release of growth factors. However, there is a third option, namely restoration of the activity of cells that have survived the injury but remain dysfunctional. Restoration of TH expression in the injured striatum after GDA^{BMP} transplantation may be an example of such a functional rescue.

Although it is tempting to speculate on the likely multiple mechanisms by which transplanted $GDAs^{BMP}$ promote functional recovery, we have tried for the sake of brevity to limit our discussion of mechanisms to the results presented in the current manuscript.

"3) An important experiment that needs to be done is to show persistence of astrocytes that were transplanted in the lesion site. Given human cells were transplanted this should be possible. MSC are not persistent and generally have transient effects and much of their effects have been related to modulation of the immune system and reduction in inflammation. It may be useful to examine if these astrocytes modulate inflammation"

Immunohistochemical staining of brain sections of animals demonstrated that transplanted $GDAs^{BMP}$ persisted in all animals, both in syngeneic rat transplants and after transplantation of human $GDAs^{BMP}$ into immunosuppressed recipients. An example has been added to SI figure 4.

As neuroinflammation has been demonstrated in PD progression, the possible effect of $GDAs^{BMP}$ on the immune response is also an important question. Our preliminary analyses of 6OHDA lesioned animals using the monocyte marker ED1 did not reveal a difference between and GDA^{BMP} treated animals in the number or morphology of monocytes recruited to the injury site.

"4) The delayed addition is a welcome strategy but I find the positive result somewhat surprising. IT suggests that TH is down regulated rather than the cells are lost to show such recovery and innervation. If the authors believe otherwise they should say so and if not they should discuss possibilities. I note their culture model should allow them to test if the conditioned medium up regulates TH expression."

We also were surprised by this outcome, and we agree that the observed recovery may also be due to a recovery of tyrosine hydroxylase expression – and by inference a recovery of TH+, dopamine

neuron function. We also agree that one possible mechanism for the action of GDA^{BMP} transplants is to restore function of cells that may still be present but have become disabled. We have added text to the discussion to clarify this interpretation.

"5) Likewise the culture experiments should be better described. One presumes that the cultures were not pure cultures but contained a mixture of astrocytes endothelial cells, microglia etc. If this is the case the others should clarify as the effect of the astrocytes could be indirect."

We apologize for not including more experimental details. Neuronal cultures were isolated from rat embryos at 18 days of gestation and plated in neurobasal medium. While we did not attempt to select for neurons or neuronal precursors, the vast majority of surviving cells (even in the positive controls) were neuronal. Most glial (including microglial cells) and endothelial cells apparently either do not adhere or survive well in these conditions. Immunofluorescent staining for GFAP, CD11b, Iba1 and ED1/CD68 revealed the following percentages of non-neuronal cells in striatal cultures: 1.1% (±0.35%) GFAP, 0.77% (±0.26%) CD11b, 1.3% (±0.32%) Iba1 and 0.6% (±0.13%) CD68 (Average of n=3, StDev).

We do not exclude the possibility that factors secreted by GDAs^{BMP} may be acting indirectly through microglial or endothelial cells. In fact, we have preliminary data suggesting that GDAs^{BMP} may promote revascularization and may also affect microglial and monocyte populations in subtle ways. However, these possibilities would simply add still more possible mechanisms that may be relevant to understanding the actions of GDAs^{BMP}. It is important to note however, that there does not appear to be any indication that we can find that promoting vascularization or modifying microglial function would have the effects we observed of promoting behavioural recovery, increasing TH expression, increasing numbers of parvalbumin+ GABAergic neurons or increasing synaptophysin levels.

6) In general when one has a nice system and sees an effect with conditioned medium one would like to see if one can block the effect of the candidate molecules or at least attempt to do so.

Dissecting the various components of GDA^{BMP} –mediated activity is important for several reasons: as a means of potentially discovering new factors, to optimize the place and time for GDA^{BMP} delivery, to perhaps discover new disease related mechanisms, to further improve the therapeutic effect, or to try and emulate the benefit of GDAs^{BMP} by using combinations of recombinant factors rather than cells. However, the strength of these cells also becomes a particular challenge: classical loss-of function experiments are difficult to do when several factors need to be tested, as disruption of just one may not have a dramatic effect.

An example is shown from our preliminary studies, in which stable, shRNA mediated knockdown was used to disrupt expression of GDNF. As shown in the figure 1 (Response to Reviewers), disruption of GDNF expression results in a reduced ability of GDAs^{BMP} to promote survival of fetal cortical neurons in vitro (**Fig 1A**, Response to Reviewer). However, GDA^{BMP}-CM still retains survival promoting activity. Another example comes from our attempt to block GSH production in GDAs^{BMP}. Here we first used the chemical inhibitor BSO, which inhibits g-glutamylcysteine synthetase (gGCS), the rate-limiting enzyme of the GSH-synthesis pathway. Here pretreatment of GDAs^{BMP} with BSO, followed by removal of BSO prior to collecting conditioned medium resulted in a near complete loss of protection of 6-OHDA treated TH+ cells, an effect which could be rescued by addition of GSH (**Fig. 1B**, Response to Reviewer). However, shRNA targeting of GCLC, the catalytic subunit of g-glutamylcysteine synthetase, had a more variable effect on the ability of GDAs^{BMP} to protect neurons in vitro (**Fig. 1C**, Response to Reviewer). These preliminary data were not included in the manuscript, as the experiments were performed using cortical neurons, not striatal TH+ neurons. In addition, it will be necessary to perform combinatorial targeting to fully dissect potential combinations of multiple factors, which – if performed in an in vivo assay – is a very ambitious undertaking.

"*7) The discussion is well written and the results carefully interpreted but I note one important comparison that is missing and that is transplant of dopaminergic neurons themselves. The rotational behaviour improvement, the recovery and the effectiveness do delayed transplants is generally better at least as reported in the literature. The authors should perhaps add that to their discussion along with a discussion of availability of their cells in numbers sufficient to move to translation.*"

We are happy to add this concern to our discussion. As the reviewer recognizes, a support cell therapy is very different from a neuronal replacement therapy.

While it is correct that our observed behavioural effects are not quite as dramatic as those that have been reported for transplantation of dopaminergic neurons in similar experiments, it is also the case that none of these other reports demonstrated a beneficial effect on PV+ interneurons or levels of synaptophysin expression.

At present the optimal source of GDAs^{BMP} is fetal derived precursors, which can readily be expanded in vitro prior to differentiation. Although we have not yet completed tests on the limits of in vitro expansion, one single isolate can readily produce one billion cells in just seven passages, and cells at seven passages retain all the phenotypic properties tested to date. In addition, we have been able to cryo-preserve differentiated GDAs^{BMP}, such that frozen cells can be thawed and transplanted without apparent loss of activity.

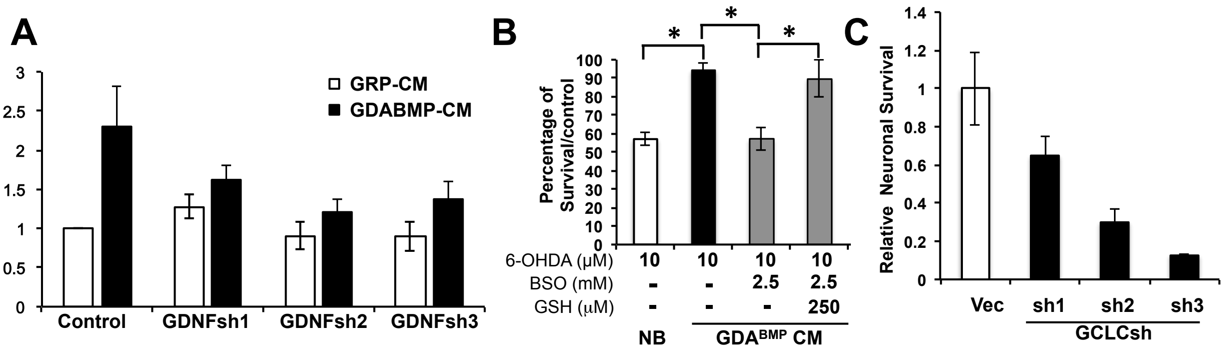

Figure 1 (Response to Reviewers): Disruption of GDNF or GCLC in GDAs^{BMP} impairs but does not abolish neuroprotective effects of GDA^{BMP}-CM. (A) shRNA mediated knock-down of GDNF reduces survival promoting effect on embryonic rat cortical neurons. (B) Chemical inhibition of GSH synthesis using BSO disrupts neuroprotective action of GDA^{BMP}-CM in cortical cultures exposed to 6-OHDA. Effect can be rescued by addition of GSH. (C) shRNA-mediated disruption of GCLC, the catalytic domain of γGCS results in reduced neuroprotection of cortical neurons exposed to 10µM 6-OHDA.

2nd Editorial Decision 12 November 2013

Thank you for the submission of your revised manuscript to EMBO Molecular Medicine. We have now heard back from the three referees whom we asked to re-evaluate your manuscript.

As you will see from the enclosed reports, while referees 1 and 3 are now supportive of publication, referee 2 remains unsatisfied, especially regarding the lack of mechanism provided in the revision.

You certainly know that it is EMBO Molecular Medicine policy to allow only a single round of revision. However, in some extreme cases, we can ask for additional experiments when they are absolutely required to make the paper stronger. We feel that this is one of those cases and we would

like to give you a last chance to address referee 2's concern and provide some understanding of how the cells have beneficial effect following damage in the CNS. We would therefore strongly encourage you to explore the avenues mentioned in the discussion section of the paper as possible mechanism.

I look forward to seeing a revised form of your manuscript as soon as possible.

***** Reviewer's comments *****

Referee #1 (Comments on Novelty/Model System):

This is an interesting paper that has been revised appropriately

Referee #1 (Remarks):

This is acceptable for publication

Referee #2 (Remarks):

I have read through the changes and edits provided in the revision.
The authors have done a number of edits that have helped clarify the paper and have added some important controls to the in vitro work. These changes have helped improve and solidify the in vitro work. However, while I appreciate the response, I still have some issues with the paper in its current form.
My issue was with the novelty of the study. Different groups using different approaches, have shown effects of various growth factors delivered by pumps, vectors or different cell lines following 6-OHDA treatment. Further, some studies have revealed behavioral benefits following these treatments. Here the authors have shown that the GDABMP cells have positive effects presumably, in part, by rescuing striatal dopaminergic neurons following 6-OHDA lesion. I agree that there are differences in the timing and transplant of this particular cell line, and in several of the measured outcomes from their previous work in the spinal cord; however, the finding that these cells have a beneficial effect following damage in the CNS is not vastly different than their previous work. It still remains to be determined how these cells mechanistically function in the CNS following damage. The discussion presents multiple avenues for how these cells may be functioning including releasing various growth factors, anti-oxidants and modulation of inflammatory responses, without a better understanding of which if any are the roles. Thus, as it stands the paper has described a nice phenotypic affect with the cells; however, the underlying mechanism remains elusive.

Referee #3 (Remarks):

The authors have responded to my concerns. I am satisfied with the changes made

Additional Author Correspondence 27 November 2013

Thank you so much for the prompt turnaround on our revised manuscript. We are delighted that two of the three referees support publication in EMM, and that all previous concerns of Referee 2 regarding the experimental design and presentation were satisfactorily addressed.

We also very much appreciate the opportunity to revise our manuscript and to address Referee 2's remaining concern regarding the novelty of the present study - an issue not shared by the other two reviewers, although perhaps the novelty of our work was not made sufficiently clear. Before revising the manuscript, however, we would like to discuss with you briefly how best to approach this.

It appears that the remaining concern of Referee 2 relates to both work by others, and our own prior discoveries. We feel, however, that the novelty of our manuscript is not infringed by other sources, as we present several novel findings that demonstrate a new approach to PD therapy:

1) Transplantation of our GDAsBMP into a neurodegenerative PD model is the first intervention to restore expression of synaptic proteins in the diseased striatum and also the first to rescue a population of GABA-ergic interneurons, both of which are impaired in PD. This is in addition to restoring tyrosine hydroxylase (TH) expression in dopaminergic neurons and improving certain behavioral outcomes, which have been the focus of previous studies,

2) We demonstrate that the delayed transplantation of this unique class of astrocytes into the already diseased striatum can promote recovery. This is unlike previous studies in which genetically-modified astrocytes were delivered prior to, or at the onset of neurodegeneration.

3) Our findings present the only current approach to PD treatment that addresses multiple critical problems at once. This has not been achieved with any prior treatment, as previous studies have focused on delivery of individual factors or the replacement of TH+ dopaminergic neurons.

We will communicate these points more clearly by revising the manuscript discussion accordingly.

Delayed transplantation of GDAsBMP into a neurodegenerative PD model also represents a completely novel and very different approach as compared to our previous studies on acute spinal cord injury (SCI). SCI and PD are two profoundly different conditions, one being an acute trauma, the other a progressive neurodegeneration. Also the affected cell types are very different: i.e. spinal neurons, oligodendrocytes and glial scar-forming astrocytes in SCI, and dopaminergic and parvalbumin+ neurons amongst others in PD. The therapeutic benefit of GDABMP transplantation into diseased striatum was in fact a surprising and unexpected result, and the experiments reported in our manuscript provide the first evidence that transplantation of this unique astrocyte population has therapeutic benefit in both acute injury and in chronic, neurodegenerative disease. In addition, while acute treatment of SCI is an experimentally useful system with limited clinical relevance, the delayed treatment of a chronic, degenerative insult, as described for the first time in our present work, is a clinically relevant intervention. We agree that both of these points could be clearer in the discussion section of the manuscript.

Referee 2 now has raised the question of mechanism, a question that was not articulated by this referee in the previous round of review. While understanding the underlying mechanisms would be interesting in some respects, such investigation is unlikely to change the main conclusions of our manuscript. Indeed, Referee 2 does not suggest that the requested experiments would have a significant impact on any of our main conclusions or on the potential medical relevance of GDABMP transplantation. Moreover, such analyses would greatly exceed the scope of the present manuscript, due to the complex nature of such studies that would require simultaneous ablation of multiple molecular mechanisms. While previous approaches, such as delivery of specific proteins or transplantation of neurons have targeted a single mechanism, the cells we have transplanted intrinsically produce multiple effector molecules, each acting in a distinct manner. We therefore suggest added discussion of these emergent properties of GDAsBMP in the revised manuscript. It is also worth noting in this context that Referees 1 and 3 did ask questions about mechanism and were fully satisfied with our response on this topic.

Taken together, we feel that the remaining criticism by Referee 2 can be readily addressed by incorporating the changes suggested above into the manuscript, particularly as Referee 2 was satisfied with all our previous revisions, and referees 1 and 3 fully support publication following previous review without the need for further experimentation.

We thank you for your time, and sincerely appreciate your help and consideration in this matter.

Additional Editorial Correspondence 28 November 2013

Thank you for your letter regarding the resubmission of your manuscript.

I have now discussed your arguments with my colleague and re-read in details the referee's comments from both rounds of review.

We feel that if you could modify the text of the article to make the novelty much clearer (as suggested in your letter) and argue the mechanism in the discussion section (also as you suggested), we would be happy to move forward. Please do resubmit your article making the changes obvious and in order to gain time, please address all editorial requirements at the same time.

2nd Revision - authors' response 13 December 2013

Thank you so much for your message. We are very grateful to you for allowing us to present our arguments, and very happy that you agreed with our suggestions. Please find enclosed the revised manuscript entitled "Delayed transplantation of precursor cell-derived astrocytes provides multiple benefits in a model of Parkinsons". In addition to the revisions made previously, we have made the suggested changes to further clarify the novelty of our work. All changes are highlighted. We have also made changes in the abstract and introduction, although the majority of modifications are in the discussion section. No changes were made in the results section. As you requested, we have also double-checked all editorial requirements.

We thank you so much for your help and feel that the manuscript has certainly improved as a result of this review process.

© EMBO
