# Supplementary material for: Delayed transplantation of precursor cell-derived astrocytes provides multiple benefits in a rat model of Parkinsons
Source: EMBO Mol Med. 2014 Jan 29;6(4):504–18. doi: 10.1002/emmm.201302878 (PMC3992077; doi:10.1002/emmm.201302878)
Supplement: Supplementary file 2 [file emmm0006-0504-sd2.pdf]

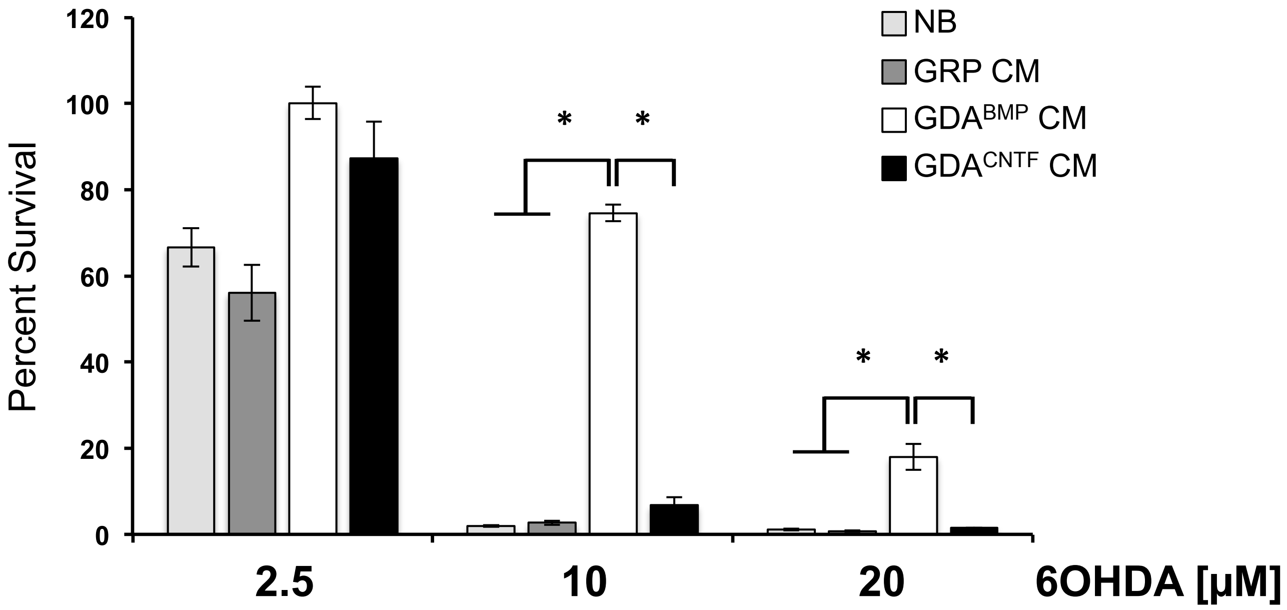

SI Figure 1: GDAs^{BMP} promote survival of cortical neurons exposed to 6-OHDA in vitro. Survival of cortical neurons in neurobasal medium (NB), GRP-, GDA^{BMP}- or GDA^{CNTF}-conditioned medium (CM) after addition of increasing doses of 6-OHDA. Mean ± S.E.M., n=3.
