# Supplementary material for: Delayed transplantation of precursor cell-derived astrocytes provides multiple benefits in a rat model of Parkinsons
Source: EMBO Mol Med. 2014 Jan 29;6(4):504–18. doi: 10.1002/emmm.201302878 (PMC3992077; doi:10.1002/emmm.201302878)
Supplement: Supplementary file 3 [file emmm0006-0504-sd3.pdf]

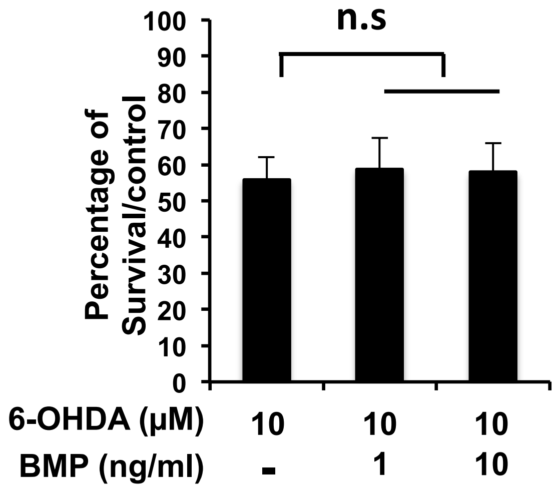


**SI Figure 2*:* BMP4 does not protect striatal TH+ neurons against 6-OHDA toxicity.** Striatal neuron cultures were prepared as in Figure 2, and treated in with 10uM 6-OHDA, with or without direct addition of BMP4. Mean ± S.E.M., n=3. *p<0.05 by ANOVA/ Bonferroni Multiple Comparison post-test.
