# Supplementary material for: Delayed transplantation of precursor cell-derived astrocytes provides multiple benefits in a rat model of Parkinsons
Source: EMBO Mol Med. 2014 Jan 29;6(4):504–18. doi: 10.1002/emmm.201302878 (PMC3992077; doi:10.1002/emmm.201302878)
Supplement: Supplementary file 5 [file emmm0006-0504-sd5.pdf]

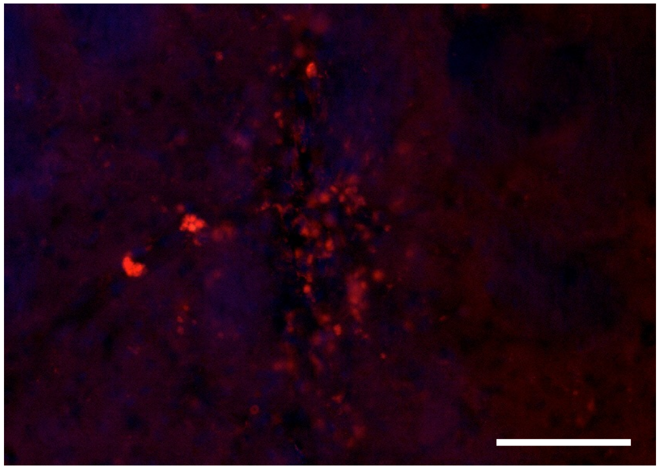

SI Figure 4: Transplanted hGDAs^{BMP} persist in the rat striatum. Human mitochondrial (hMA) staining of transplanted cells. Scale bar 100nm.
