# Supplementary material for: Delayed transplantation of precursor cell-derived astrocytes provides multiple benefits in a rat model of Parkinsons
Source: EMBO Mol Med. 2014 Jan 29;6(4):504–18. doi: 10.1002/emmm.201302878 (PMC3992077; doi:10.1002/emmm.201302878)
Supplement: Supplementary file 6 [file emmm0006-0504-sd6.pdf]

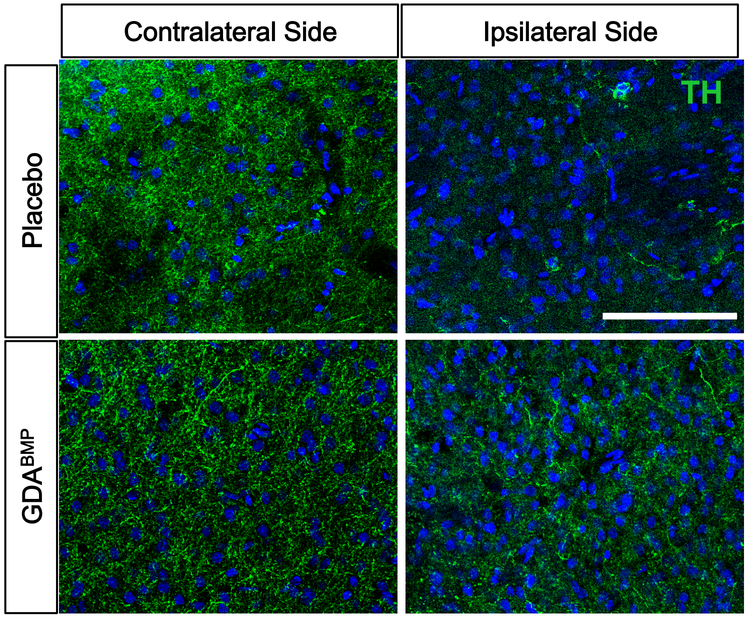


**SI Figure 5: GDAs$^{BMP}$ promote recovery of tyrosine hydroxylase expression in 6-OHDA lesioned animals.** Tyrosine hydroxylase (TH) staining in matched sections of the ipsi and contralateral striatum of placebo and GDA$^{BMP}$ treated animals. Blue channel in shows DAPI counterstaining. Scale bar = 100 mm.
