# Supplementary material for: Delayed transplantation of precursor cell-derived astrocytes provides multiple benefits in a rat model of Parkinsons
Source: EMBO Mol Med. 2014 Jan 29;6(4):504–18. doi: 10.1002/emmm.201302878 (PMC3992077; doi:10.1002/emmm.201302878)
Supplement: Supplementary file 7 [file emmm0006-0504-sd7.pdf]

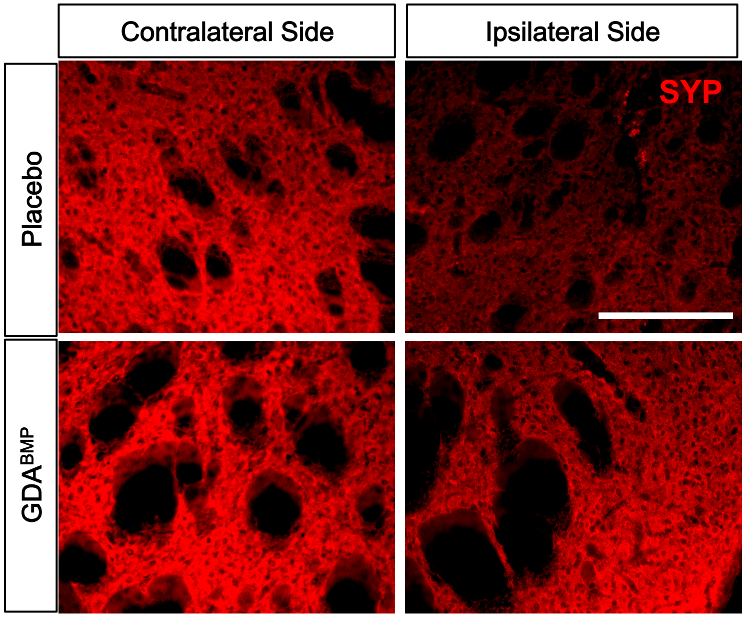

SI Figure 6: GDAs^{BMP} promote recovery of synaptophysin expression in 6-OHDA lesioned animals. Synaptophysin (SYP) staining in matched sections of the ipsi and contralateral striatum of placebo and GDA^{BMP} treated animals. Blue channel shows DAPI counterstaining. bar = 100 mm.
