# Supplementary material for: Delayed transplantation of precursor cell-derived astrocytes provides multiple benefits in a rat model of Parkinsons
Source: EMBO Mol Med. 2014 Jan 29;6(4):504–18. doi: 10.1002/emmm.201302878 (PMC3992077; doi:10.1002/emmm.201302878)
Supplement: Supplementary file 8 [file emmm0006-0504-sd8.pdf]

Table of Contents

- Figure 1: GDAs^{BMP} promote survival of cortical neurons exposed to 6-OHDA in vitro. Page 1
- Figure 2: BMP4 does not protect striatal TH+ neurons against 6-OHDA toxicity. Page 2
- Figure 3: Experimental time line of in vivo experiments. See material and methods for details of procedures. Page 3
- Figure 4: Transplanted hGDAs^{BMP} persist in the rat striatum. Page 4
- Figure 5: GDAs^{BMP} promote recovery of tyrosine hydroxylase expression in 6-OHDA lesioned animals. Page 5
- Figure 6: GDAs^{BMP} promote recovery of synaptophysin expression in 6-OHDA lesioned animals. Page 6
